# Association Between Fermented Food Consumption and Sleep Quality Under Psychological Stress: Prospective Cohort Study

**DOI:** 10.1002/fsn3.70537

**Published:** 2025-07-07

**Authors:** Maria Dobielska, Natalia Bartosik, Ksawery Olczyk, Adrian Suława, Mateusz Litwin, Jakub Parys, Michał Seweryn Karbownik

**Affiliations:** ^1^ Department of Infectious, Tropical, and Parasitic Diseases for Children Władysław Biegański Voivodeship Specialist Hospital Łódź Poland; ^2^ Department of Psychiatry Ludwik Rydygier Voivodeship Integrated Hospital Toruń Poland; ^3^ Institute of Sociology, University of Lodz Łódź Poland; ^4^ Ziemo‐Vita Medical Center Wrocław Poland; ^5^ University Clinical Hospital No. 2 of the Medical University of Lodz Łódź Poland; ^6^ Central Clinical Hospital of Medical University of Lodz Łódź Poland; ^7^ Department of Pharmacology and Toxicology Medical University of Lodz Łódź Poland

**Keywords:** brain‐gut axis, fermented food, food record, insomnia, probiotics, sleep quality

## Abstract

Sleep quality might be dependent on the gut‐brain axis that employs gut microbiota. As, in adulthood, diet has the greatest influence on microbiota, the aim of the study was to investigate whether the consumption of fermented food was associated with sleep quality among medical students under exam stress. A prospective cohort questionnaire‐based study was conducted online on a group of medical students under exam stress from the Medical University of Lodz, Poland. The participants were asked to provide information regarding their health and sleep quality (Pittsburgh Sleep Quality Index, PSQI) in the last week before the exam. During the 4 days before the exam, the participants were recording the consumption of various types of food and beverages, including fermented food. The study sample was 280 medical students (their mean age was 22.63, 172 were female). Students whose fermented food consumption was found in the second tercile had higher sleep quality under stress (mean PSQI 5.13) than students in the first (mean PSQI 5.73) and third (mean PSQI 6.17) terciles of fermented food consumption (*p* = 0.021). There was no association between the fermented food consumption and gastrointestinal symptoms under stress, even though, in general, the more symptomatic the students were, the worse sleep quality they had. Nonlinear association between fermented food consumption and sleep quality sets thinking about the complexity of biological mechanisms in which gut microbiota could possibly impact sleep under stress. The influence of widely available fermented food on sleep requires further study.

## Graphical abstract

A prospective cohort questionnaire‐based study was conducted online on a group of 280 Polish medical students under exam stress to assess the association between their fermented food consumption and sleep quality. The association was found to be V‐shaped. Both raw and adjusted analyses showed that students whose fermented food consumption was found in the second tercile had lower mean PSQI scores, which indicated higher sleep quality, than students in the first and third terciles.

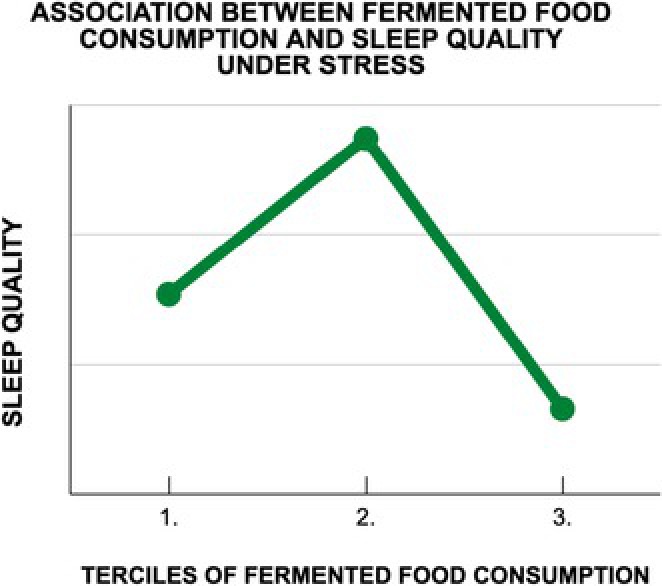

## 1 Introduction

The Father of Medicine Hipocrates was known to emphasize the role of sleep by saying: “in whatever disease sleep is laborious, it is a deadly symptom; but if sleep does good, it is not deadly”. Nowadays, sleeping difficulties are no longer considered primary predictors of life or death. However, the benefits of good sleep quality have been broadly proved (Stern 2021). Among others, it straightens somatic health by preventing cardiovascular diseases (Tobaldini et al. 2017) and supporting the immune system (Besedovsky et al. 2019). Moreover, in line with healthy sleep, goes correct emotional regulation (Walker and van der Helm 2009). Not surprisingly, sleep disturbances are highly prevalent among patients suffering from major psychiatric diseases (Mairinger et al. 2023).

Taking into account that healthy sleep seems to maintain the body and the mind in an overall well‐being state, it is worth considering how to assure it. Sleep quality was proved to depend among the others on changes in the hypothalamus‐pituitary–adrenal axis (Steiger 2007; Van Reeth et al. 2000) and neuroimmune mechanisms (Imeri and Opp 2009). In turn, these systems stay in the strong relation of bilateral regulation with the brain‐gut axis (Martin et al. 2018). The brain‐gut axis employs the central nervous system, the neuroendocrine and neuroimmune systems, the autonomic nervous system, the enteric nervous system, and the gut microbiota (Bermúdez‐Humarán et al. 2019). Some interventions that regulate the gut microbiota were found to alleviate depression and anxiety (Liu et al. 2019) and improve sleep quality (Irwin et al. 2020). The examples of such interventions are supplementation with probiotics, prebiotics, or providing fermented food in the diet. According to the recent meta‐analysis of eleven clinical randomized controlled trials on the effect of probiotics and paraprobiotics (inactivated probiotics) on patients with sleep disorders and subhealthy sleep conditions, probiotic supplementation improved sleep states to some extent in adults with sleep disorders and healthy adults with condition‐induced sleep disorders (Yu et al. 2024), what is mechanistically explained elsewhere (Haarhuis et al. 2022).

However, unlike the well‐defined and commercially available probiotic preparations, fermented food is much more accessible and widely used around the world (Tamang et al. 2020), even if they are heterogeneous and do not necessarily contain probiotic bacteria. For instance, in Eastern Europe, fermented food still plays an essential role in traditional cuisine or even represents a domestic strategy of health care (Sõukand et al. 2015). Therefore, it seems to be an issue of great importance to assess if fermented food is as favorable as expected by the societies in terms of sleep quality. Not all the published results were in line—clinical assessment of consumers of fermented food revealed, for example, increased symptoms of depression (M. Karbownik et al. 2022; Yu et al. 2018; X. Zhang et al. 2021) and anxiety (M. Karbownik et al. 2022) or negligible effect on cognition (Marx et al. 2020). As both anxiety and depression are bidirectionally associated with insomnia (Blake et al. 2018), these results drive scientific curiosity towards the link between the fermented food and sleep, especially during stressful and cognitively demanding times, which appear common these days and may further impair sleep quality.

Moreover, a diet abundant in fermented food could also trigger plenty of gastrointestinal disturbances, since fermentation in the guts leads to among others gaseous distension of the intestine. Being affected by for example fluctuance, abdominal pain or diarrhea might lead to poor sleep (Spiller 2021). On the other hand, some probiotic bacteria, which can be provided in fermented food, were proved to have prominent efficacy in relieving the symptoms of patients with irritable bowel syndrome (T. Zhang et al. 2022). In reaction to stress, also people who do not meet the diagnostic criteria for irritable bowel syndrome experience similar gastrointestinal symptoms as the ill (Zia et al. 2022). Therefore, those who consume more fermented food in a stressful period might also provide in such a way relief to their gut and, as a result, better sleep may occur among them. Nevertheless, this proposal needs broader scientific investigation.

Medical students are particularly exposed to stress, not only due to high academic demands, but also because of direct contact with human tragedies which are undoubtedly associated with illnesses. It might explain the high number of depressive and anxiety disorders as well as sleep disturbances in this group (Karbownik, Mokros, and Kowalczyk 2022).

The aim of the present study was to investigate whether the consumption of fermented food was associated with sleep quality among medical students under exam stress. Moreover, if such an association existed, the aim was also to assess if it was linked to gastrointestinal symptoms.

## 2 Materials and Methods

The STROBE (STrengthening the Reporting of OBservational studies in Epidemiology) guidelines for reporting cohort studies were applied to outline the study description (STROBE_checklist_v4_cohort.Pdf n.d.).

### 2.1 Study Design

The study was approved by the Bioethics Committee of the Medical University of Lodz, Poland (RNN/65/22/KE, received on 08 March 2022). It was mandatory from the volunteers to express their informed consent in an electronic manner to take part in the study.

A prospective cohort study was conducted on a group of third‐year medical students of the Medical University of Lodz, Poland, under exam stress. The study was questionnaire‐based and was carried out online. Firstly, the participants were asked to complete a survey (Survey 1, Step A), which included questions about sociodemographic data, chronotype, personality traits, and health‐related information. Then, the participants were recording consumption of selected food and beverages starting from 3 days before the exam day until the exam itself (Step B). The upcoming subject exam served as a real‐life model of stress, as used in our previous studies (Karbownik, Mokros, and Kowalczyk 2022; Karbownik, Mokros, Dobielska, et al. 2022; Tokarek et al. 2023). A few hours before the exam, students filled in another survey (Survey 2, Step C) to provide information about sleep quality, physical activity, general quality of diet, and gastrointestinal disturbances in the last week before the exam.

**FIGURE 1:**
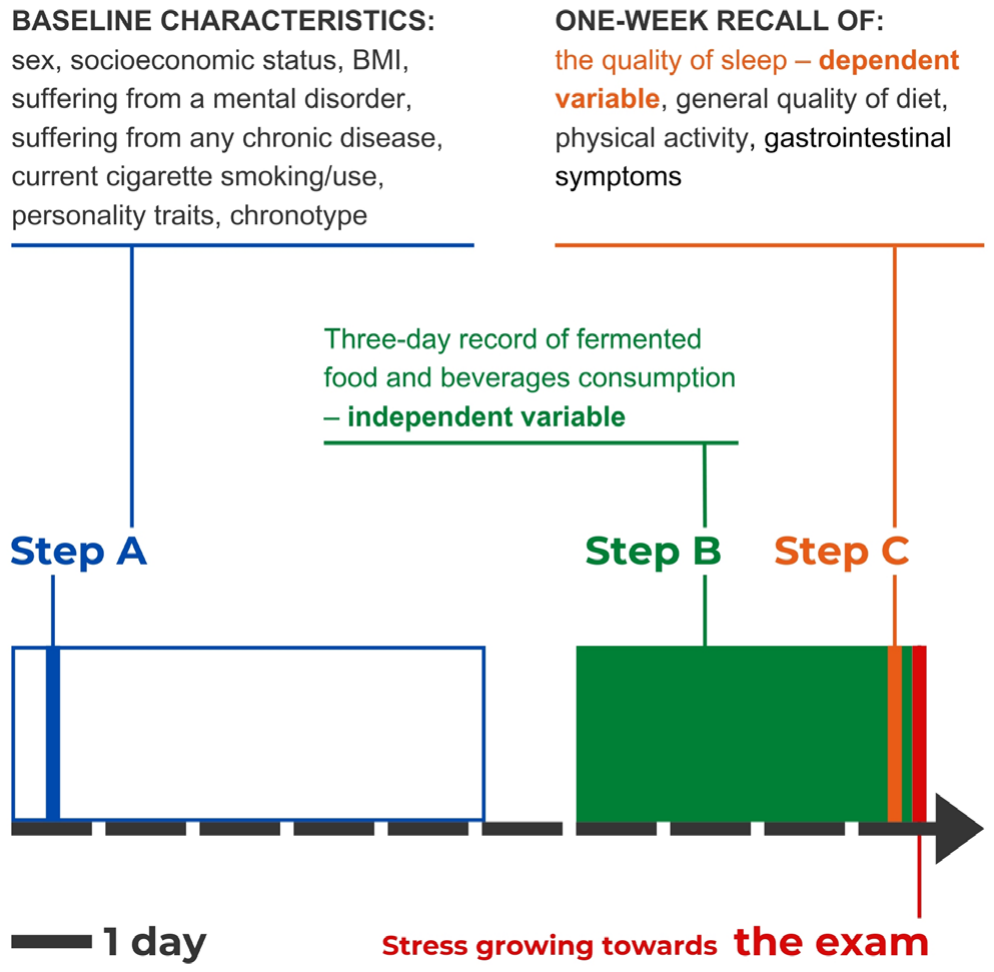
The study design. Step A stands for carrying out Survey 1, Step B stands for providing records of food and beverages, Step C stands for carrying out Survey 2. Independent and dependent were written in color, confounding variables were written in black.

The study timeline is presented in Figure 1.

### 2.2 Setting

All data was collected through surveys. Participants independently completed the surveys, with no direct oversight by the research team. The surveys were constructed in Google Forms and shared with the participants by Microsoft Teams (MT) to be filled out in particular time frames. Surveys allowed for no forced answering. Students were gradually reminded about the upcoming research steps by short posts on the MT group. Illustrated information bulletins, pictorial instructions, and study calendar were also shared. To minimize the risk of response bias and to prevent the participants from changing their habits during the study, especially in matters of diet, they were informed only about the very general aim of the study—association between selected everyday behaviors and psychometric indexes of mental health. The issue of fermented food was not mentioned towards the participants.

#### 2.2.1 Step A

Step A aimed to assess baseline characteristics of the participants. Survey 1 (which content is presented in the Supporting Information S1–S3.) was shared. Participants were given 5 days (from 15/06/2022 to 19/06/2022) to fill out the survey (Figure 1).

Survey 1 gathered the following information:

- Chronotype—assessed by the Chronotype Questionnaire (CQ) (Oginska 2011).
- Expression of five personality traits—assessed by the Big Five Inventory‐Short questionnaire (BFI‐S) (Brust et al. 2016; Hahn et al. 2012).
- Health‐related features, such as:
  ○ General quality of diet in past few months—assessed by the Starting The Conversation (STC) (Paxton et al. 2011),
  ○ Suffering from chronic diseases,
  ○ Cigarettes smoking and e‐cigarettes using,
  ○ Physical activity,
  ○ Socioeconomic status,
  ○ Respondent's particulars—such as gender, age, body‐mass index (BMI).

#### 2.2.2 Step B

The objective of Step B was to gather information about almost four‐day (from 21/06/2022 until the exam on 24/06/2022) consumption of fermented food and beverages among the medical students in stressful conditions. In order to keep the participant blinded towards the aim of the study, the questions about fermented food were hidden among many questions about common food products. For these needs the food record (FR) was used (which content is available in the Supporting Information 2). FR was a complete research tool created by the authors, successfully used in previous, similar studies (Karbownik, Mokros, and Kowalczyk 2022; Karbownik, Mokros, Dobielska, et al. 2022; Tokarek et al. 2023). The questionnaire was provided in an electronic version, adapted also for use on mobile phones.

The participants were asked to record the mass of every single piece of food and beverage, which was listed in FR. The duration of the records lasted almost 4 days, as it started 3 days before the exam day and it also included almost the whole exam day, because students were examined in the evening (Figure 1). Due to the fact that students took the exam at different times, they had individual times of records—each student's consumption was recorded until her or his exam time. The participants were obligated to send FR after every single meal or beverage, but the minimal accepted number of FR was one per day.

#### 2.2.3 Step C

On the exam day (24/06/2022), a few hours before the exam (Figure 1.), when stress seemed to approximate its peak, the participants were asked to fill in Survey 2 (Supporting Information S1–S3.), which mainly aimed to gather information about their functioning in stressful conditions.

Questions about the following issues were asked:

- The quality of sleep in the last week and last month before the exam—Pittsburgh Sleep Quality Index (PSQI) was used to evaluate each period (Buysse et al. 1989).
- Diet‐related matters:
  ○ General quality of diet in 1 week before the exam—assessed by STC questionnaire (Paxton et al. 2011), used also in Step A.
  ○ Gastrointestinal symptoms in 1 week before the exam—assessed by a 10‐items questionnaire with three‐point Likert scale designed by the authors.
- Intensity of physical activity in the last week before the exam—assessed by International Physical Activity Questionnaire (IPAQ) (Lee et al. 2011).
- Additional questions:
  ○ The intake of the first generation antihistamine agents or systemic antimicrobial agents, the hospitalization, the exacerbation of chronic illnesses in the past 4 weeks before the exam.

Polish adaptations of the questionnaires originally available in English (BFI‐S (Strus and Cieciuch 2021), PSQI (*Kwestionariusz Jakości Snu Pittsburgh (Pittsburgh Sleep Quality Index—PSQI)* n.d.), IPAQ (Biernat et al. 2007)) were used. CQ (Kontrymowicz‐Ogińska 2011) was designed in Polish and its original version was employed in the study, but its English version (Oginska 2011) was used for descriptions in the manuscript.

#### 2.2.4 Exam

Final exam in Pharmacology served as a model of real life stress, as it was one of the most demanding obligations during the whole medical studies. The exam lasted 60 min, contained 60 multiple‐choice questions, and was carried out on computers. Such examinations had triggered typical stress effects on bodies (Koudela‐Hamila et al. 2022) and they had been comparable on this matter to commonly used laboratory stress protocols (Henze et al. 2017). The research team was not involved in the organization of the exam.

### 2.3 Participants

Third‐year medical students from the academic year 2021/22, who met the formal criteria to sit for the exam in Pharmacology in the first attempt, were all invited to the study. There were no specific exclusion criteria from the study in order not to reveal the aim of the study towards the participants. The participants who declaredthe intake of the first‐generation antihistamine agents or systemic antimicrobial agents, the hospitalization, or the exacerbation of chronic illnesses in the past 4 weeks before the exam were allowed to participate, but their data were excluded from the statistical analysis.

### 2.4 Variables

The variables were graphically presented in Figure 1. The analysis between fermented food consumption in almost 4 days before the exam (independent variable) and sleep quality in 7 days before the exam (dependent variable) was performed. To calculate the independent variable, all masses in grams of fermented food recorded in FR throughout almost 4 days before the exam and on the exam day before the exam were summed up and divided by the number of days. Due to the fact that the students took the exam at different times, the time of records was individual. The dependent variable was expressed as Global PSQI score, and it was calculated according to the 1‐week PSQI, which was described in detail below in the “Data measurements” section.

The potential confounders included in the analyses were sex, socioeconomic status, BMI, suffering from a mental disorder, suffering from any chronic disease, physical activity (IPAQ), general diet quality (STC) and gastroenterological symptoms in 1 week preceding the exam, current cigarette smoking/use, each of five personality traits (BFI‐S) and chronotype. One of the potential confounders—the gastrointestinal symptoms—was also included in facultative analyses as a non obligatory confounder.

### 2.5 Data Measurements

#### 2.5.1 Step A

In the Survey 1 the following research tools were used to measure:

- Chronotype—assessed by CQ, that aims to describe two dimensions of chronotype: subjective phase, that is, morning–evening preference and subjective amplitude, that is, distinctness of the diurnal rhythm of activation. The questionnaire contains two subscales: morningness‐eveningness scale (8 items) and distinctness scale (6 items). Participants are asked to indicate the extent to which they agree with these statements in a three‐point Likert scale (from 1 to 3). The points from the items related to particular subscales are summed up. In morningness‐eveningness scale is possible to achieve from 8 to 40 points and the result is categorized into: extreme early bird (8–15 points), moderate early bird (16–20), middle type (21–27), moderate night owl (28–32), extreme night owl (33–40). In the distinctness scale it is possible to achieve from 6 to 30 points and the result is categorized into: little distinct rhythm (6–15), averagely distinct rhythm (16–22), very distinct rhythm (23–30) (Kontrymowicz‐Ogińska 2011).
- Expression of five personality traits—assessed by BFI‐S, in which five dimensions of personality are evaluated; participants are asked to express the extent to which they agree with 15 statements in a seven‐point Likert scale. For each of five dimensions of personality 3 statements are assigned. Intensification of each of personality traits is presented numerically in the range of 3–21. The more points, the more intense the trait is (Strus and Cieciuch 2021).
- Health‐related features, such as
  ○ General quality of diet in the past few months—assessed by STC questionnaire which is an 8‐items simplified food frequency instrument enabling a brief dietary assessment. Each question is rated on a scale (from 0 to 2), where higher scores indicate less healthy eating habits. Overall diet quality is expressed as the sum of all points in the range from 0 (maximally healthy diet) to 16 (maximally unhealthy diet), with the midpoint of 8 (Paxton et al. 2011),
  ○ Suffering from chronic diseases, especially mental disorders and usage of psychotropic drugs—assessed by 4 questions designed by the authors,
  ○ Cigarettes smoking and e‐cigarettes using—assessed in 2 questions designed by the authors,
  ○ General physical activity in the past few months—assessed in the single‐item five‐point semantic differential scale from 1 (“I have no physical activity at all”) to 5 (“I do sport intensively 5 times a week”), with the midpoint of 3, designed by the authors.
- Socio‐economic status—assessed by six multiple choice questions designed to the needs of this study by one of the authors, a sociologist. As social sciences gather knowledge through surveys with complex sets of questions, it was a difficult task to try to assess the social position of the respondents without dominating the whole survey with questions about their socioeconomic background. Simultaneously, the social position seemed to be a confounding variable in the research, so it was crucial to determine it. Finally, a simplified research tool based on a short set of questions was found to express the social position as a continuous variable. Three factors, equal toward each other, were taken into account: father education, mother education, and the place of living. The more educated a parent was and the bigger the population of the place of residence, the higher the social position. Each of these variables was converted by the researchers into points from 0 to 2, which allowed them to place students on the scale from 0 to 6. Moreover, the students were also asked to subjectively assess their social position on a scale from 1 to 10, where 10 means the highest. Four classes were distinguished based on this assessment: low (1–5), lower‐middle (6), upper‐middle (7), high (8–10). Such division was proposed due to the relatively unequal distribution of the responses. It seems that there was a tendency of the respondents to define their social position around the “well‐understood” middle. This means that, as a rule, in this type of question, a respondent is likely to choose the number 6 or 7, as long as the person does not live with the conviction that his or her life situation is drastically different from others.

#### 2.5.2 Step B

Food record questionnaire used in Step B (Supporting Information 2) was created to record the consumption of fermented food. To minimize a response bias, the vast majority of the questions were related to the food products that were not fermented, but very common in everyday middle‐european diets.

Food record contained six general categories of food products, such as (1) meat, sausages, fish, seafood, (2) dairy and eggs, (3) grain products, (4) vegetables, (5) fruits and nuts, (6) sweets. Fermented food and beverages were hidden within these categories and they were represented by: cheese, yogurt, kefir, soured milk, kvass and unpasteurised beer, pickled cucumbers and their pickling juice, sauerkraut and its pickling juice and other fermented vegetables and their pickling juice.

The participants were asked to express the mass of food in grams. FR was designed to help the users with accuracy—the questionnaire was richly illustrated on photos presenting regular portions of food, captioned by their mass in grams. The photos were copied from a website providing professional dietary service (*IleWazy.Pl—Baza Produktów Spożywczych i Zestaw Narzędzi Przydatnych Przy Gotowaniu i Dietach* n.d.). Moreover, the survey did not accept letters, but only numbers.

This tool had been successfully used in our previous research (Karbownik, Mokros, and Kowalczyk 2022; Karbownik, Mokros, Dobielska, et al. 2022; Tokarek et al. 2023). Recent validation showed that mean error was −0.0 g (95% CI −2.9 to 2.9), suggesting no systematic error and its high accuracy (*p* = 0.99, one‐sample *t* test); some imprecision of the FR measure was detected as mean relative absolute error was 16.7% (95% CI 13.5% to 20.0%) (Tokarek et al. 2023).

#### 2.5.3 Step C

In the Survey 2, the following research tools were used to measure:

- Sleep quality, evaluated by PSQI. This self‐report questionnaire is composed of 19 items to evaluate seven components of sleep quality: subjective sleep quality, sleep latency, sleep duration, habitual sleep efficacy, sleep disturbances, use of sleeping medication, and daytime dysfunction. The level of disturbance in each component is expressed by one of four ordinal numbers (from 0 to 3). The bigger the number is, the more disturbed each component of sleep quality. The sum of all seven components expresses total sleep quality, which means the extent to which the sleep is overly disturbed (Global PSQI score). Global PSQI score is presented in the range from 0 to 21, where the bigger the number, the more disturbed sleep is. The Global PSQI score > 5 had been established to distinguish poor sleepers and potential clinical sleep issues. PSQI regards the period of 4 weeks (Ong and Suh 2013). In our study, PSQI was adapted to assess 1 week preceding the exam because it was a critical period in the research. To validate such a method, we investigated if there was a correlation between the results of the original PSQI and the PSQI regarding 1 week. Students' sleep quality expressed as Global PSQI Score in the last week before the exam was positively correlated with their sleep quality in the last 4 weeks before the exam (*r* = 0.765, *p* < 0.0001).
- Gastrointestinal symptoms—assessed by a 10‐items questionnaire with three‐point Likert scale designed by the authors. The severity of each of 10 symptoms was expressed in the scale from 0 (lack of the symptom) to 2 (severe symptom), with the midpoint of 1. Whereas the overall intensity of gastrointestinal symptoms, expressed as the sum of all 10 single symptoms, was presented in the scale from 0 (lack of such symptoms) to 20 (when each of the symptoms is severe), with the midpoint of 10.
- Intensity of physical activity—assessed by IPAQ, a 10‐items tool that helps to estimate how much time, calculated in minutes, an individual spent on vigorous or moderate physical activity, as well as on walking and sitting (10). Results can be reported as a continuous score—MET minutes per week. MET stands for metabolic equivalent of task. MET minutes per week are calculated as MET level multiplied by minutes of activity per day multiplied by days per week. MET level is a variable that differs depending on the intensity of physical activity and amounts to 3.3 per walking, 4.0 per moderate intensity, and 8.0 per vigorous intensity. Therefore, the more MET minutes per week a respondent achieves, the greater his or her physical activity is. Three levels of physical activity are proposed: low (no activity is reported OR some activity is reported but not enough to meet Categories moderate or high), moderate (3 or more days of vigorous activity of at least 20 min per day OR 5 or more days of moderate‐intensity activity and/or walking of at least 30 min per day OR 5 or more days of any combination of walking, moderate‐intensity or vigorous‐intensity activities achieving a minimum of at least 600 MET‐minutes/week) and high (vigorous‐intensity activity on at least 3 days and accumulating at least 1500 MET‐minutes/week OR 7 or more days of any combination of walking, moderate‐ or vigorous‐intensity activities accumulating at least 3000 MET‐minutes/week).

### 2.6 Bias

Solid precautions were made to address potential sources of bias. To reduce performance bias and to prevent the participants from the Hawthorne effect, they were not informed that the research aimed to measure their fermented food consumption. The questions about fermented food were hidden among questions about common dietary products of all categories. The participants were informed that the study aimed to assess the association between selected everyday behaviors and psychometric indices of mental health. Moreover, to prevent the study participants from revealing the aim of the study during its course, the participants who met the exclusion criteria were also allowed to finish the study, but their data were later excluded from the analyses. In order to address recall bias students were encouraged to fill out the FR questionnaire often, ideally after every meal, by short posts and self‐made memes related to the study, published in MT. Validated questionnaires were used to assess the majority of study variables to reduce interviewer bias and response bias. The data collection was conducted within strict, predetermined time frames to minimize the risk of publication bias.

### 2.7 Study Size

In order to demonstrate the effect size of β regression coefficient of 0.2 with a statistical power of 0.8 and statistical significance of 0.05, minimal sample size was estimated to be 193, as indicated by G*Power, version 3.1.9.2 (Faul et al. 2007). Assuming no more than 20% of drop‐outs and no more than 20% of participant records being excluded due to above‐mentioned reasons, the goal was to recruit at least 301 volunteers.

### 2.8 Quantitative Variables

Fermented food consumption was categorized into three groups. The data (of each person) were ordered according to the value of the fermented food consumption expressed in grams. Two cut‐point values (the so‐called tercile cut‐off points) of fermented food intake were identified, dividing the sample into three groups equally represented by 33.3% of individuals each. The first tercile group represents 33.3% of the population with the lowest fermented food intake (an intake smaller or equal to the first cut‐off value); the second tercile represents 33.3% of the population with the middle intake (greater than the first cut‐off value, smaller or equal to the second cut‐off value) and the third tercile group represents 33.3% of the population with the highest intake (an intake greater than the second cut‐off value). These groups were treated as categorical variables in the analyses. Chronotypes were also treated as categorical variables. All the other variables were treated as continuous and included in the analysis as linear.

### 2.9 Data Analysis

There was little missing data in the datasheet (461 missing data values for a total of 120,780 values, that is, 0.38%). Assuming a random pattern of missingness, the missing data was imputed with multiple imputation by chained equations (MICE) using R software version 4.0.0 (package “mice” version 3.8.0; R Foundation for Statistical Computing).

Descriptive statistics included mean with standard deviation (SD) for continuous variables, median with 1st and 3rd quartiles for ordinal or skewed variables and number with frequency for categorical variables. General linear modeling was used to assess the differences in sleep quality between the terciles of fermented food consumption. When the difference occurred to be significant, the contrast analysis was performed to diagnose linear trend (−1, 0, 1) and V‐shaped trend (1, −2, 1) between fermented food consumption and sleep quality. The raw analyses were supplemented with covariate‐adjusted with potential confounders linearly linked to the outcome. *p*‐value below 0.05 was considered statistically significant. STATISTICA 13.3 (Statsoft; Tulsa, OK, USA) was used in data analysis.

## 3 Results

### 3.1 Participants

Four hundred sixty‐one students participated in the study, but 358 of them (77.7%) completed it by filling out the Survey 1, Survey 2 and at least one FR per day and only they were considered. Out of these 358 participants those who declared the following events in 4 weeks prior to the study: the intake of the first generation antihistamine agents or systemic antimicrobial agents, the hospitalization, the exacerbation of chronic illnesses, were excluded from the statistical analyses, therefore the final number of the participants included in the analyses amounted to 280 (78.2% of those who completed the study).

### 3.2 Baseline Characteristics of the Participants

According to participants' reports, the mean age of them was 22.63 [standard deviation (SD) = 1.29] and the majority of them were female (*n* = 172, which is 61.4%). Their mean BMI was 22.49 (SD = 3.15), which is found within the normal range. However, over one fourth of them (*n* = 74, 26.4%) were overweight or obese. The majority of students had both parents with higher education and their place of residence was cities. The social position of the study participants was assessed as high, with its middle value amounted to 5 in 0–6 scale. It was in line with a subjective assessment of living standards that showed that over 80% of the participants defined themselves as the representatives of the middle or higher class.

Only about one third of the participants (*n* = 99, 35.4%) did not suffer from any chronic disease. The most common were allergic diseases and mental disorders. The group of students with mental health problems amounted to 49. In this group were students who declared to have a mental disorder (*n* = 44, 15.7%) or students who admitted that they were taking psychotropic drugs during the course of the research (*n* = 40, 14.3%). Personality traits of the participants as well as their health‐related behaviors in the last few months, such as general quality of diet, physical activity, and cigarette smoking/use, were described in Table 1.

**TABLE 1:** Basal characteristics of the participants, from Survey 1.

Characteristics	Mean (standard deviation), median (1st–3rd quartiles) or absolute number (frequency)
**Age**	
[years]	22.63 (1.29), 22.0 (22.0–23.0)
**Sex**	
Female	172 (61.4%)
Male	108 (38.6%)
**Socioeconomic status** ^a^	
*Father's education*	
Primary education	3 (1.1%)
Secondary education	99 (35.4%)
Higher education	178 (63.6%)
*Mother's education*	
Primary education	2 (0.7%)
Secondary education	61 (21.8%)
Higher education	217 (77.5%)
*Place of residence*	
City with population over 200,000	108 (38.6%)
City with population under 200,000	119 (42.5%)
Village	53 (18.9%)
Social position according to parents' education and place of residence^a^	4.59 (1.21) 5 (4–6)
*Self‐assessed social position*	
High class	67 (23.9%)
Upper‐middle class	99 (35.3%)
Lower‐middle class	59 (21.1%)
Low class	55 (19.6%)
**Anthropometry**	
Body‐mass index [kg/m^2^]	22.49 (3.149) 22.31 (20.06–24.62)
**Morbidity**	
Any chronic disease	99 (35.4%)
Allergic diseases	66 (23.6%)
Mental disorders	44 (15.7%)
Endocrine diseases	22 (7.9%)
Gastroenterological diseases	13 (4.6%)
Immunological diseases	7 (2.5%)
Cardiological diseases	7 (2.5%)
Cancerous diseases	2 (0.7%)
Oral cavity diseases	4 (1.4%)
Neurological diseases	1 (0.4%)
**Health‐related behaviors in the last few months**	
General physical activity^b^	2.84 (1.05) 3 (2–4)
General quality of diet^c^	8.73 (2.70) 9 (7–11)
Current cigarettes smoking/use	55 (19.6%)
**Personality traits** ^d^	
Neuroticism	13 (10–16)
Extraversion	12 (9–15)
Openness	14.5 (12–17)
Agreeableness	15 (12–17)
Conscientiousness	16 (13–18)

*Note:* The instruments, used to assess these features, were thoroughly described in the section “Data measurements, Step A” of Materials and Methods.

^a^ Social position in a 0–6 scale, where 0 stands for the lowest and 6 for the highest.

^b^ Physical activity in a 1–5 scale, where 1 stands for “I have no physical activity at all” and 5 “I do sport intensively 5 times a week”.

^c^ General quality of diet assessed by STC in a 0–16 scale, where 0 stands for maximally healthy diet and 16 for maximally unhealthy diet, with the midpoint of 8.

^d^ Intensification of each personality trait assessed by BFI‐S in a 3–21 scale, where the bigger the number, the more intense the trait was.

**FIGURE 2:**
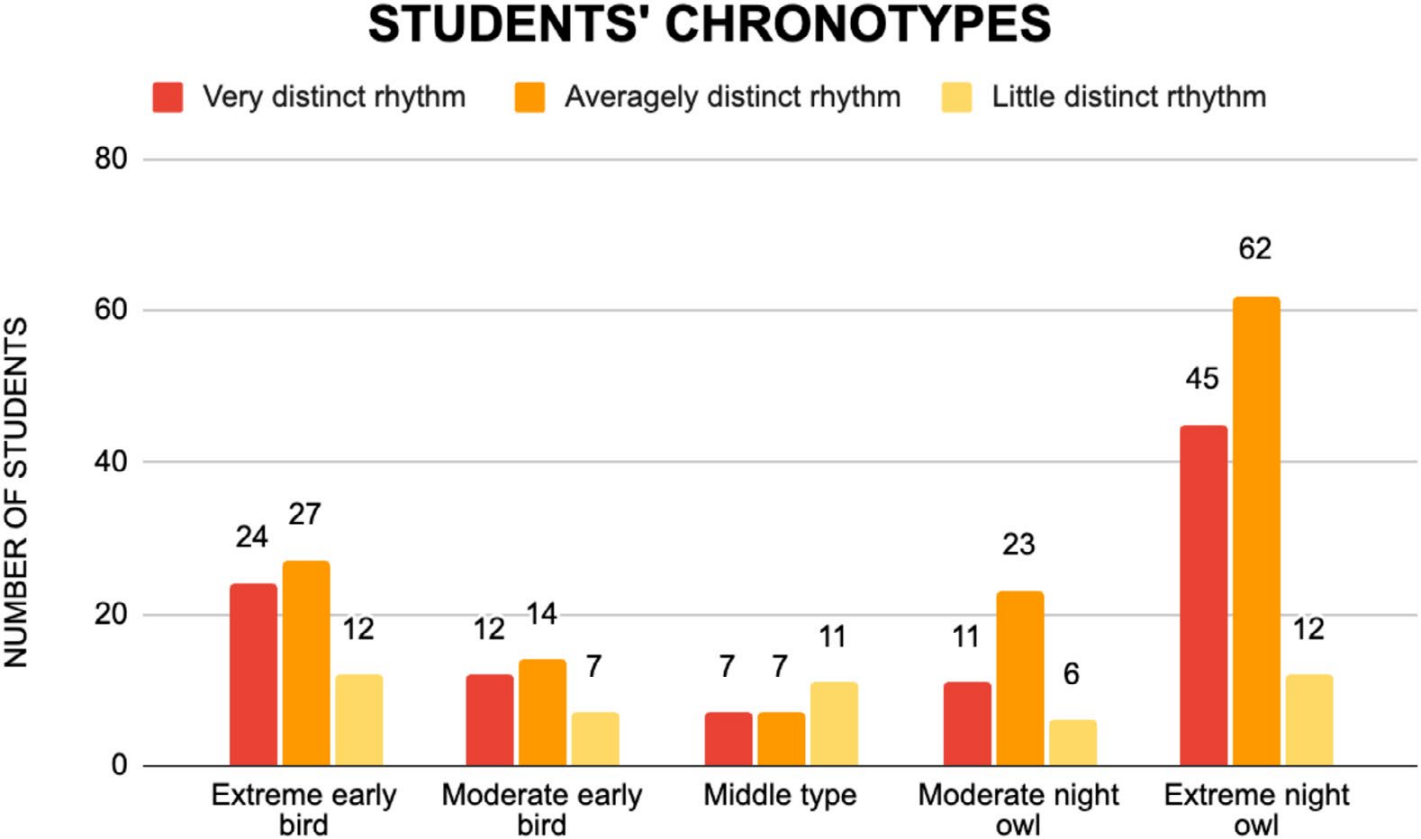
Students' chronotypes according to the Chronotype Questionnaire.

Almost a half of students (*n* = 119) were found to be extreme night owls, which meant they tended to be most active and alert in the evening. Among these subjects, such rhythm was well distinctive. About one‐fifth of the students (*n* = 63) presented an adverse chronotype—extreme early bird—that made them to be most active and alert in the morning. Three intermediate chronotypes—moderate early bird, middle type and moderate night owl—were less represented and in the first two of them the rhythm was rather blurred. The details were presented in Figure 2.

### 3.3 Health‐Related Parameters in the Week Preceding the Stressful Event

Students' habits in the week preceding the exam remained similar in comparison to the last few months. Their quality of diet, assessed by STC questionnaires, appeared unchanged during the course of the study (Wilcoxon signed‐rank test, *p* = 0.34).

Students experienced some sleep disturbances along the way to the exam. The most significant was *daytime dysfunction* and low *subjective sleep quality*. However, even these two the most affected components of sleep quality were still below the midpoint (1.5, “the lower value, the better sleep quality”). Nevertheless, the mean sum of all components, which expressed mean total sleep quality (mean Global PSQI score), amounted to 5.75, which was enough to indicate poor sleepers among the students (ranged from 5 to 21) in 1 week timeframe (Ong and Suh 2013). Global PSQI scores of 181 (64.6%) of the participants were found within the range from 5 to 21.

The vast majority of subjects (97.5%) experienced some gastrointestinal disturbances. The most common symptom was nausea (86.8%) and least was vomiting (2.5%). About one‐third of the study group declared to feel abdominal pain, belching, flatulence or fullness. However, the average intensity of such disturbances was low (3.86 out of 20). Details are presented in Table 2.

**TABLE 2:** Health‐related parameters of the participants in the week processing the final exam. GI, gastrointestinal.

Parameter	Mean (standard deviation)	Median (1st–3rd quartiles)
**Health related behaviors**		
Physical activity [MET‐minutes/week]^a^	1396 (1344)	974 (406–1931)
Quality of diet^b^	8.58 (2.54)	9 (7–11)
**Sleep quality** ^**c**^		
Subjective sleep quality	1.14 (0.64)	1 (1–2)
Sleep latency	1.08 (0.99)	1 (0–2)
Sleep duration	0.91 (0.82)	1 (0–2)
Habitual sleep efficacy	0.30 (0.62)	0 (0–0)
Sleep disturbances	0.87 (0.50)	1 (1–1)
Use of sleeping medication	0.25 (0.77)	0 (0–0)
Daytime dysfunction	1.21 (1.01)	1 (0–2)
Total sleep quality (Global PSQI score)	5.75 (2.84)	5 (4–7)

	Absolute number (frequency)	Intensity of GI symptoms	
		Mean (standard deviation)	Median (1st–3rd quartiles)
**GI symptoms in 1 week preceding the stressful event** ^**d**^			
Intensity of GI symptoms^**d**^		3.86 (3)	3 (1–6)
Any GI symptom	273 (97.5%)		
Nausea	243 (86.8%)	0.91 (0.35)	1 (1–1)
Vomiting	7 (2.5%)	0.03 (0.16)	0 (0–0)
Belching	89 (31.8%)	0.45 (0.62)	0 (0–1)
Heartburn	39 (13.9%)	0.2 (0.46)	0 (0–0)
Regurgitation	38 (13.6%)	0.19 (0.45)	0 (0–1)
Abdominal pains	77 (27.5%)	0.48 (0.68)	0 (0–1)
Diarrhea	66 (23.6%)	0.34 (0.57)	0 (0–1)
Constipation	30 (10.7%)	0.16 (0.43)	0 (0–0)
Flatulence	84 (30%)	0.56 (0.71)	0 (0–1)
Stomach fullness	96 (34.3%)	0.56 (0.68)	0 (0–1)

*Note:* The instruments, used to assess these features, were thoroughly described in the section “Data measurements, Step C” of Materials and Methods.

^a^ Moderate pattern of physical activity according to IPAQ, which means 5 or more days of any combination of walking, moderate‐intensity or vigorous intensity activities achieving at least 600 MET‐minutes/week, but no more than 3000 MET‐minutes/week.

^b^ General quality of diet assessed by STC in a 0–16 scale, where 0 stands for maximally healthy diet and 16 for maximally unhealthy diet, with the midpoint of 8.

^**c**^ Sleep quality assessed by PSQI in which the bigger the number, the more disturbed each value in a 0–3 scale for a particular component and in a 0–21 scale for Global PSQI score (the sum of all components). Global PSQI score (total sleep quality) > 5 had been established to distinguish poor sleepers.

^**d**^ GI symptoms in a 0–2 scale for a particular symptom and 0–20 scale for all of them, where the bigger the number, the more intense the symptom is and “0” stands for lack of symptom.

### 3.4 Eating Habits in 4 Days Preceding the Stressful Event

The mean daily intake of fermented food was 102.48 g. The most represented group of fermented products were yogurts, kefir, sour milk and sour cream (mean 220.03 g per day) and the last were pickled vegetables, other than cucumbers and cabbage (mean 6.94 g per day).

Detailed data are presented in Table 3.

**TABLE 3:** Consumption of the analyzed food products per day in a period of almost 4 days preceding the exam. The instruments, used to assess the above parameters, were thoroughly described in the section “Data measurements, Step B” of Materials and Methods.

Fermented food products [gram/day]		
Food product	Median (1st–3rd quartiles)	Mean (standard deviation)
Cheese	46.17 (24.46–81.03)	56.78 (43.95)
Yogurts, kefir, sour milk, sour cream	195.48 (98.35–313.69)	220.03 (158.7)
Unpasteurised kvass and beer	0 (0–16.45)	36.1 (83.79)
Pickled cucumbers	32.28 (0–65.24)	43.00 (46.77)
Pickled cabbage	0 (0–26.08)	15.43 (28.94)
Other pickled vegetables	0 (0–0)	6.94 (21.57)
Sum of fermented food products	90.92 (49.03–143.8)	102.48 (68.33)
	1st tercile 63.927, 2nd tercile 151.18	

### 3.5 Association Between Consumption of Fermented Food and Quality of Sleep Under Psychological Stress

**FIGURE 3:**
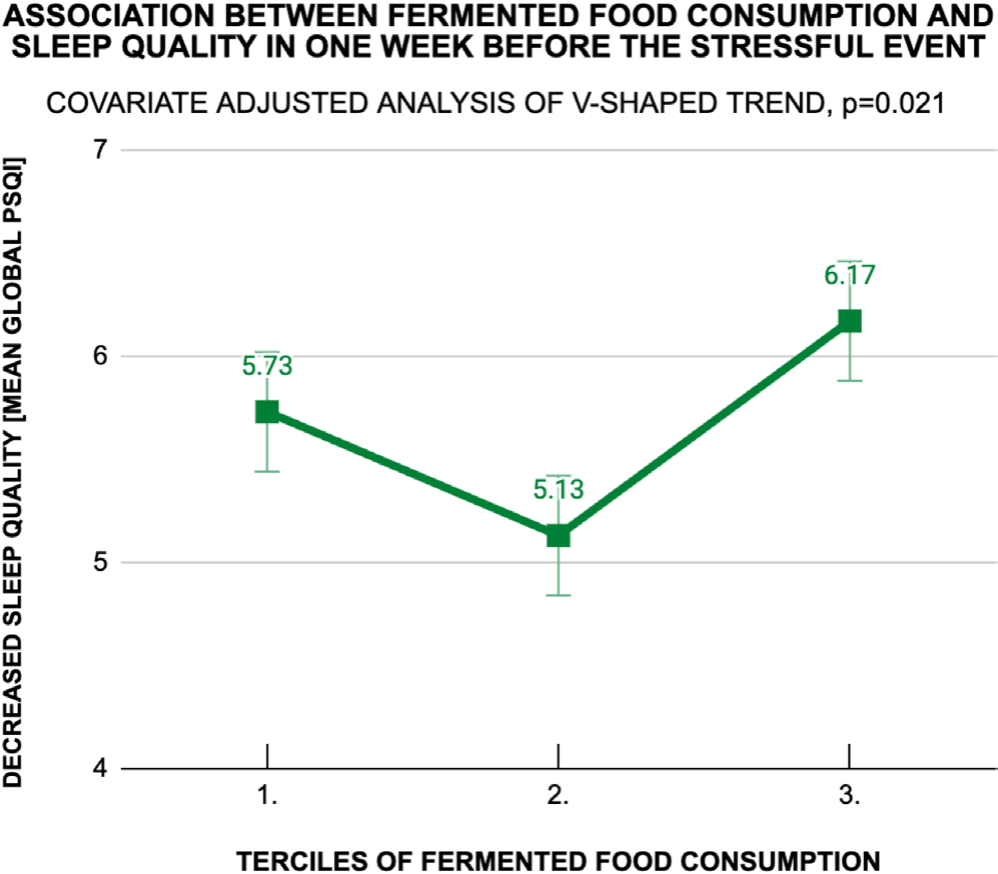
Covariate adjusted analysis of V‐shaped trend in the association between fermented food consumption and sleep quality in the 1 week before the stressful event. The higher Global PSQI score, the worse sleep quality. In the raw analysis of the same association, the curve was very close to the presented and also V‐shaped. PSQI, Pittsburgh Sleep Quality Index.

There was a significant difference in sleep quality (Global PSQI score) between three terciles of fermented food consumption—in raw (*F* [2277] = 3.14, *p* = 0.045) and adjusted analyses (*F* [2260] = 3.22, *p* = 0.042). However, there were no significant differences in sleep quality between the terciles of fermented food consumption in the raw and adjusted analyses of an increasing linear trend (*p* = 0.15 and *p* = 0.28, respectively). Not only total sleep quality (Global PSQI score), but also all sleep components were tested, but all the associations were insignificant. Nevertheless, the raw and adjusted analysis of the nonlinear, V‐shaped trend showed significant association for sleep quality (*p* = 0.041 and *p* = 0.021, respectively)—students, whose fermented food consumption was found in the second tercile, yielded lower Global PSQI score, than students from the first and third terciles, which meant the sleep quality of the former group was higher (Figure 3.) These associations were not found significant for any of the sleep components, neither in the raw nor in adjusted analysis of V‐shaped trend, except for “use of sleeping medication”. The highest load of the total score appeared from “use of sleeping medication”, “sleep latency” and “sleep duration”, as reflected in the analysis of V‐shaped trend for each sleep component. Details are presented in Table 4.

**TABLE 4:** Association between consumption of fermented food and quality of sleep under psychological stress (*n* = 280). Statistically significant results are shown in bold.

Quality of sleep (Global PSQI score)	Terciles of fermented food consumption (grams)					
	Increasing linear trend					
	Raw analysis			Adjusted analysis^a^		
	Contrast estimate		*p*	Contrast estimate		*p*
	Point	95% CI		Point	95% CI	
Total quality of sleep	0.59	−0.22, 1.40	0.15	0.44	−0.36, 1.25	0.28
Sleep components						
Subjective sleep quality	0.04	−0.14, 0.23	0.66	0.06	−0.12, 0.24	0.51
Sleep latency	0.06	−0.22, 0.35	0.67	−0.04	−0.33, 0.24	0.76
Sleep duration	0.09	−0.15, 0.32	0.47	0.04	−0.21, 0.29	0.75
Habitual sleep efficacy	0.11	−0.06, 0.29	0.21	0.09	−0.09, 0.27	0.33
Sleep disturbances	0.07	−0.07, 0.21	0.37	0.10	−0.04, 0.25	0.18
Use of sleeping medication	0.06	−0.15, 0.28	0.59	0.08	−0.15, 0.30	0.51
Daytime dysfunction	0.16	−0.13, 0.45	0.28	0.12	−0.16, 0.40	0.40

Nonlinear, V‐shaped trend						
	Raw analysis			Adjusted analysis^a^		
	Contrast estimate		*p*	Contrast estimate		*p*
	Point	95% CI		Point	95% CI	
Total quality of sleep	**1.47**	**0.06, 2.87**	**0.041**	**1.65**	**0.25, 3.05**	**0.021**
Sleep components						
Subjective sleep quality	−0.13	−0.44, 0.19	0.42	−0.12	−0.43, 0.19	0.45
Sleep latency	0.43	−0.07, 0.92	0.09	0.33	−0.16, 0.83	0.19
Sleep duration	0.34	−0.06, 0.75	0.10	0.37	−0.06, 0.80	0.10
Habitual sleep efficacy	0.24	−0.06, 0.55	0.12	0.11	−0.21, 0.43	0.49
Sleep disturbances	0.09	−0.16, 0.34	0.50	0.17	−0.09, 0.43	0.21
Use of sleeping medication	**0.42**	**0.04, 0.8**	**0.029**	**0.50**	**0.11**, **0.89**	**0.013**
Daytime dysfunction	0.07	−0.43, 0.57	0.78	0.29	−0.21, 0.77	0.25

^a^ Adjusted for sex, socioeconomic status, BMI, suffering from a mental health illness, suffering from any chronic disease, physical activity (IPAQ), general diet quality (STC) and gastroenterological symptoms in the 1 week preceding the exam, current cigarette smoking/use, each of the five personality traits (BFI‐S), and chronotype (CQ).

### 3.6 Association Between the Fermented Food Consumption and the Presence of Gastrointestinal Symptoms Under Psychological Stress

There were insignificant differences between the terciles of fermented food consumption in intensity of gastrointestinal symptoms under stress in raw and adjusted analyses; similarly, insignificant increasing linear trends and V‐shaped trends were found. The differences in ten gastrointestinal symptoms between the terciles of fermented food consumption were also insignificant. The following confounders were included in the adjusted analysis: sex, socioeconomic status, BMI, suffering from a mental health illness, suffering from any chronic disease, physical activity (IPAQ), general diet quality (STC), current cigarette smoking/use, and each of five personality traits (BFI‐S). Details are presented in Supporting Information 3.

### 3.7 Coassociation Between Fermented Food Consumption, Intensity of Gastrointestinal Symptoms and Quality of Sleep

**FIGURE 4:**
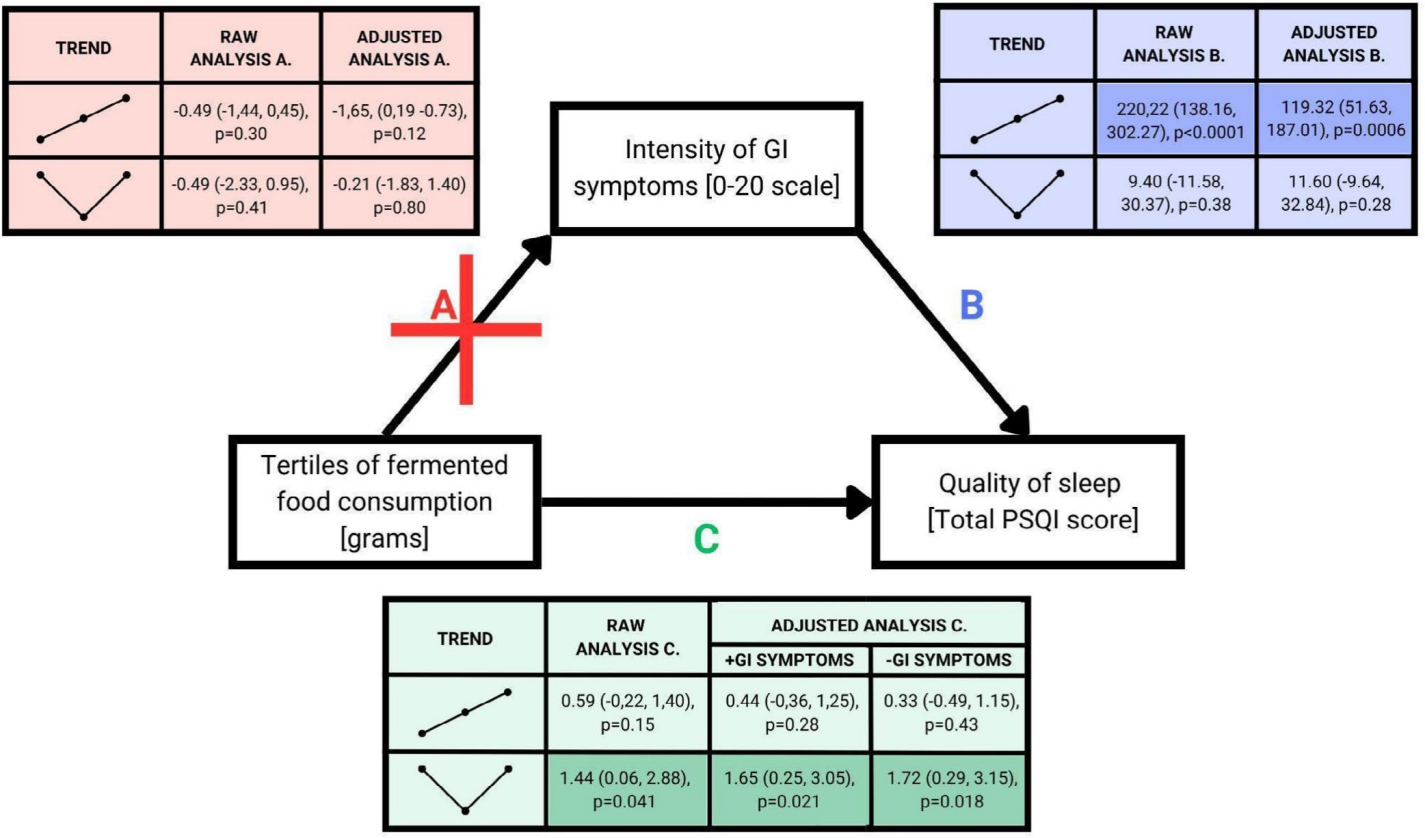
Coassociations between fermented food consumption, intensity of GI symptoms, and quality of sleep. The results were presented in a particular order: Point estimate (confidence interval), *p* value. Statistically significant associations were presented on darker backgrounds. For the purpose of examining trends between the intensity of GI symptoms and quality of sleep, the former was treated as categorical, and the contrasts were as following −8, −7, −6, −5, −4, −3, −2, −1, 1, 2, 3, 4, 5, 6, 7, 8 for linear trend and as following 3, 2, 1, 0, 0–1, −2, −3, −3, −2, −1, 0, 0, 1, 2, 3 for V‐shaped trend. GI, gastrointestinal, PSQI, Pittsburgh Sleep Quality Inventory. The analyses C were adjusted for potential confounders as follows: Sex, socioeconomic status, BMI, suffering from a mental health illness, suffering from any chronic disease, physical activity (IPAQ), general diet quality (STC), current cigarette smoking/use, each of five personality traits (BFI‐S), chronotype (CQ) and alternatively for gastroenterological symptoms in 1 week preceding the exam. Whereas in the analyses A and B, the potential confounders were the same except for chronotype (CQ) and gastrointestinal symptoms in 1 week preceding the exam that were excluded. Moreover, in the analyses B, which regarded a 1‐week long period of subacute stress, general diet quality (STC) in 1 week preceding the exam was a potential confounder.

Fermented food consumption predicted the quality of sleep in either raw and adjusted analyses of nonlinear, V‐shaped trends. Such results occurred also when the intensity of gastrointestinal symptoms was not treated as one of the potential confounders. Fermented food consumption was not linked to the intensity of gastrointestinal symptoms. However, the intensity of gastrointestinal symptoms was associated with the quality of sleep in either raw or adjusted analysis of increasing linear trend, but not V‐shaped trend. Details are presented in Figure 4.

## 4 Discussion

### 4.1 Non Linear, V‐Shaped Association Between Fermented Food Consumption and Quality of Sleep Under Stress

Medicine should always try to address social problems, as they are at the core of health problems. While facing social inequalities in access to health care, diet modifications seem more accessible than medical care, medication, or dietary supplements. According to our research, easily available fermented food was associated with changes in sleep quality under psychological stress, possibly through the gut‐brain axis. If this association is confirmed, it would open the area for new strategies for cheap domestic care. In our research, both raw and adjusted analyses showed that students from the second tercile of fermented food consumption yielded lower PSQI total scores, than students from the first and third terciles, which meant the sleep quality under psychological stress of the former group was higher. Surprisingly, there was no association between the fermented food consumption and gastrointestinal symptoms under psychological stress, even though the more symptomatic the students were, the worse sleep quality they had. The nonlinear association between fermented food consumption and sleep quality and lack of association between fermented food consumption and gastrointestinal symptoms sets thinking about the biological mechanism standing behind it.

Sleep and gut microbiota are inextricably intertwined and gut microbiota seem to be modulated by diet. The majority of data indicate that proper microbes in the gut could elicit better sleep (Ito et al. 2024; Yu et al. 2024), which could explain why in our research students who consumed the average amount of fermented food achieved better sleep. The composition of microbes of the gastrointestinal tract exhibit circadian rhythm and their metabolism fluctuates in response to the daily feeding/fasting schedule (Matenchuk et al. 2020). In the study on mice it was found that gut microbial metabolites, such as the short‐chain fatty acids butyrate and acetate, directly modulate circadian clock gene expression, therefore they regulate circadian rhythm (Leone et al. 2015). Maintained circadian rhythm brings healthy sleep (S.‐Y. Sun and Chen 2022). Moreover, gut microbes are responsible for neurotransmitters' production—bacteria regulate colonic enterochromaffin cells, the main producers of serotonin in the human body and they produce small amounts of serotonin themselves (Matenchuk et al. 2020). Serotonin, together with norepinephrine, are present at higher levels during wakefulness. Moreover, serotonin is a precursor for melatonin, a hormone that regulates the sleep–wake cycle (Matenchuk et al. 2020). Fermented food could also impact sleep through hormonal pathways via its impact on stress hormones of the hypothalamus‐pituitary–adrenal (HPA) axis. Gut microbiota plays an important role in the development and regulation of the HPA axis. The intensity of stress response, understood as a release of the hormones of the HPA, depends on gut microbiota composition and to some extent is vulnerable to its changes (Sudo 2016; Sudo et al. 2004). Another area that connects gut microbiota with sleep is the immune system. The gastrointestinal tract with its microbiota is considered the largest immunological organ in the human body having a central role in regulating immune homeostasis (Takiishi et al. 2017). Sleep deprivation leads to an inflammatory response (Matenchuk et al. 2020), while some inflammatory cytokines were found to be involved in sleep phases regulation in mice (Fang et al. 1998; Nguyen et al. 2019). The complex relationship between the gut microbiota, circadian rhythm, eating habits and sleep seems to be generally bilateral because sleep deprivation led to aberrant microbiota diurnal fluctuations (Thaiss et al. 2014), dysbiosis (Poroyko et al. 2016; J. Sun et al. 2023; Thaiss et al. 2014; Yang et al. 2023), bacterial translocation across the gut barrier (Summa et al. 2013; Yang et al. 2023) as well as increased appetite and food intake (Gomes et al. 2023). Therefore, both the intuitive direction of causality (where gut microbiota may affect sleep quality) and the reverse direction appear plausible, based on the Bradford Hill criteria, which have become the most widely cited framework for causal inference in epidemiologic studies (Fedak et al. 2015). In this context, the criteria of specificity and biological gradient are not met, and the criterion of plausibility does not preclude bidirectional causality.

Taking into consideration all above data, a diet rich in fermented food rather seems to have the real potential to improve sleep quality. However, in our study, only the students, whose fermented food consumption was found in the middle range, slept better than others. It leads to the consideration, that probably among the students whose consumption was very little, the intake of fermented food could have been insufficient to bring the beneficial effect. In turn, the students who ate a lot of fermented food could have been exposed to a bigger risk of contact with pathogenic microbes and their toxins. So far, pathogenic bacteria, fungi, and viruses have been found multiple times in fermented food, especially in the Global South, where access to clearwater is limited and work conditions are poor (Skowron et al. 2022). It could have been expected that the increasing dose of fermented food would be associated with the growing incidence of gastrointestinal symptoms, as it was found in other studies (Baron et al. 2024; Galena et al. 2022). In our research, there was no such association. It could have been caused by the fact that almost every student (97.5%) had experienced at least one disturbing symptom of the gastrointestinal tract in the stressful week preceding the exam. As the students who had been hospitalized, treated with systemic antimicrobial agents or who had exacerbation of chronic illnesses were excluded from the analysis, therefore the probable cause of gastrointestinal disturbances seemed to be somatization in reaction to psychological stress. Too many students experienced gastrointestinal symptoms to clearly analyze the association. It is advisable for further studies on cohorts not exposed to stress.

The results of our research seem to be in line with other clinical studies on this topic. In adulthood, diet has the greatest influence on microbiome, and its composition promotes the growth of particular taxa (David et al. 2014). Numerous studies on humans provided promising data about the association of probiotics intake with the improvement of sleep parameters. Five meta‐analyses of clinical trials indicated that probiotics supplementation significantly improved sleep quality of adults (Chu et al. 2023; Irwin et al. 2020; Ito et al. 2024; Santi et al. 2023; Yu et al. 2024). Among others, it was a very similar study to ours (Nishida et al. 2019), in which medical students under psychological stress due to a cadaver dissection course were given *Lactobacillus gasseri* CP2305, what brought a positive effect on their sleep in comparison to the placebo group. However, one meta‐analysis of clinical trials revealed that gut microbiota modulation was not associated with significant improvement in sleep quality (Gil‐Hernández et al. 2023). Further analyses direct scientific attention towards relevant studies about depression because depression and insomnia have similar pathophysiology and often cooccur (Baglioni et al. 2011). There was similar research in which the association between adherence to a healthy diet and the occurrence of depression was also found nonlinear and V‐shaped (Sánchez‐Villegas et al. 2015). Sánchez‐Villegas et al. proved that better adherence to healthy dietary patterns was associated with a reduced risk of depression among Spanish adults. However, data suggested a threshold effect so that although the risk of depression was reduced when comparing moderate versus lower adherence to healthy dietary patterns, there was not much extra benefit for the comparison between moderate and high or very high diet quality scores (Sánchez‐Villegas et al. 2015). The trend was the same in our study in terms of the amount of consumed fermented food. The authors (Sánchez‐Villegas et al. 2015) concluded that when all the essential nutritional needs had been met, there had been no further advantage from further adherence to healthy dietary patterns. They paid special attention to the supply of omega‐3 acids in depression prevention. Omega 3‐acids are the products of the metabolism of gut microbiota. Moreover, healthy eating behaviors proposed in their study included increased consumption of vegetables, fruits, and whole‐grain bread—among these products are prebiotics, nondigestible food ingredients that promote the growth of beneficial microorganisms in the intestines. It seems that the most beneficial to health might be to find a golden mean in dietary choices.

This study has its strengths and limitations and should be interpreted based on them. Firstly, the study could have been affected by information bias and response bias due to self‐reporting data collection. In terms of some variables, the participants were asked to recall the routine and repeatable events from the past, which could increase the risk of recall bias. Moreover, sleep quality was not measured by objective methods, such as polysomnography. Instead, the subjective, but popular PSQI questionnaire was used, and it was modified to relate to a 1‐week period. The modification that was made to relate to a 1‐week timeframe instead of the original 4 weeks requires comment. A similar methodological approach has been used in other studies to assess mood in terms of possible depressive symptoms. The most popular questionnaires about depressive symptoms (Patient Health Questionnaire‐9, Beck Depression Inventory‐Second Edition) that also have diagnostic value regard the period of 2 weeks (BDI‐II, n.d.; PHQ9_Polish for Poland.Pdf, n.d.). However, tools such as The Depression, Anxiety and Stress Scale‐21 Items questionnaire were designed to measure the emotional states of depression, anxiety, and stress over the past week (DASS‐21.Pdf, n.d.), whereas pictograms allow for assessing current mood (M. S. Karbownik and Hicks 2022). Therefore, the usage of the PSQI questionnaire to assess sleep quality in the last week before the stressful event, not to diagnose insomnia, seems to be a rational choice. Moreover, the PSQI questionnaire was used to assess 3‐week‐long periods in a similar study about the impact of probiotics supplementation on students' sleep quality (Marotta et al. 2019). Nevertheless, the validated FR questionnaire definitely strengthens the methodology—dietary records provided the best estimates of absolute dietary intakes and outperformed food frequency questionnaires (Park et al. 2018). Moreover, the analyses were adjusted for carefully selected confounders, which also made it more accurate. However, another area that needs a comment is the method of assessment of gastrointestinal symptoms. The original questionnaire was used instead of validated tools for a few reasons, for the most part, in order to prevent satisficing behavior of the respondents. Firstly, due to the fact that the validated questionnaires were longer, they could have discouraged participants who were under stress and time pressure from accurately answering (Koloski et al. 2017; Kulich et al. 2008). They were also directed to patients, not to future health professionals, who knew the meaning of the symptoms (Kulich et al. 2008). Moreover, even when a validated questionnaire was technically shorter, it needed to be filled out once per day, which would have resulted in a much greater required effort for the participant than the tool applied in our study (Walter et al. 2021).

The next important limitation of the study is convenience sampling. Our study was conducted on a nonrandom sample, which prevents us from straightforward generalization. However, as the members of the sample share some distinctive demographic characteristics, the results can serve as a basis for the formulation of new hypotheses regarding the demographic group of Polish medical students under stress. The results cannot be generalized to Polish medical students unexposed to stress. Moreover, our study does not allow us to reason about causality between the study variables. Nevertheless, it can lay the groundwork for further research, especially in terms of the influence of widely available fermented food on mental health disorders, which is definitely needed. Fermented food consumption seems to be a more important source of gut microbiota modification than probiotics because it is widely available, commonly used, tasty, and there is a strong tradition of its consumption. Therefore, it is worth knowing what impact it makes.

## 5 Conclusions

In Polish medical students under stress, fermented food consumption may be associated with quality of sleep and such association seems to have a V‐shaped pattern—students who eat an average amount of fermented food appear to have better sleep quality. Fermented food consumption may be insignificantly associated with gastrointestinal symptoms severity, which in turn seems to be linearly linked to sleep quality—sleep quality decreases with an increasing severity of gastrointestinal symptoms. Further studies are needed to confirm our findings, to examine causal relationships and biological mechanisms behind these phenomena.

## Author Contributions

**Maria Dobielska:** conceptualization (equal), data curation (equal), formal analysis (lead), investigation (lead), methodology (equal), project administration (lead), visualization (lead), writing – original draft (lead), writing – review and editing (equal). **Natalia Bartosik:** formal analysis (supporting), investigation (equal), methodology (supporting), writing – review and editing (equal). **Ksawery Olczyk:** investigation (supporting), methodology (equal), writing – review and editing (equal). **Adrian Suława:** investigation (equal), writing – review and editing (equal). **Mateusz Litwin:** investigation (supporting), methodology (equal), resources (supporting), writing – review and editing (supporting). **Jakub Parys:** investigation (supporting), methodology (equal), resources (supporting), writing – review and editing (supporting). **Michał Seweryn Karbownik:** conceptualization (lead), data curation (equal), formal analysis (supporting), funding acquisition (lead), investigation (supporting), methodology (equal), project administration (supporting), resources (equal), supervision (lead), validation (equal), writing – review and editing (equal).

## Conflicts of Interest

The authors declare no conflicts of interest.

## Supporting information


Appendices S1–S3.

## Data Availability

The data that support the findings of this study are openly available in Mendeley Data repository at http://doi.org/10.17632/zpx4wjy4pf.1.
