# Supplementary material for: Association Between Fermented Food Consumption and Sleep Quality Under Psychological Stress: Prospective Cohort Study
Source: Food Sci Nutr. 2025 Jul 7;13(7):e70537. doi: 10.1002/fsn3.70537 (PMC12234256; doi:10.1002/fsn3.70537)
Supplement: Supplementary file 1 — Appendices S1–S3. [file FSN3-13-e70537-s001.docx]

**Supplementary Material 1.**

English version of the original surveys designed in Polish:

**Survey 1**

Dear Student,

We invite you to participate in the study titled *"Assessment of the Relationship Between Selected Health Behaviors and Psychometric Indicators of Mental Health".* Participation is voluntary, and you can withdraw at any stage of the study without providing a reason or facing any consequences. The participation is anonymous. Among the personal data, we only ask for your student ID number, which will allow us to match your responses from different surveys to each other. Other personal data available in the academic system (such as name and surname) will not be accessible to the researchers. The study has been approved by the Bioethics Committee of the Medical University of Łódź.

If you have any questions regarding the study, please direct them to Maria Dobielska (via private chat or a chat dedicated to study participants on MS Teams, or email: mariadobielska@gmail.com).

Before we officially begin the study, we kindly ask you to provide your consent to participate in the study below.

**Informed and Voluntary Consent**

- I have read the Information to the Study Participant.
- The researcher has provided me with comprehensive answers to my questions, and I am sufficiently informed about the course of the study.
- I meet the inclusion criteria for the study: I am a third-year medical student at the Medical University of Łódź, and I have no formal obstacles preventing me from taking the final pharmacology exam in the first term.
- I give my informed and voluntary consent to participate in the study titled *"Assessment of the Relationship Between Selected Health Behaviors and Psychometric Indicators of Mental Health."* Participation will involve completing two electronic surveys and an electronic dietary record. I understand that I can withdraw my consent at any time without facing any consequences. I agree to the processing of my personal data in accordance with the data protection law.

If you have read the above information and provided your consent to participate in the study, click "Next" to begin.

**Welcome to the study!**

You are now an official Participant in the Study! Congratulations on joining this elite group!

Ahead of you lies Survey 1. In this survey, we will ask questions about yourself, your circadian rhythm, and your coping mechanisms for stress. There will also be some questions about your diet, physical activity and medications. Completing this survey will take approximately 15-20 minutes. Let's get started!

Please write down your student ID number, which was assigned to you as a student of the Medical University of Łódź.

**You**

To what extent do the following statements describe you? For each statement, indicate how much you agree with this.

I see myself as someone who…

- worries a lot,

strongly disagree/ disagree/ somewhat disagree/ neither agree nor disagree/ somewhat agree/ agree/ strongly agree

- gets nervous easily,

strongly disagree/ disagree/ somewhat disagree/ neither agree nor disagree/ somewhat agree/ agree/ strongly agree

- remains calm in tense situations,

strongly disagree/ disagree/ somewhat disagree/ neither agree nor disagree/ somewhat agree/ agree/ strongly agree

- is talkative,

strongly disagree/ disagree/ somewhat disagree/ neither agree nor disagree/ somewhat agree/ agree/ strongly agree

- is outgoing, sociable,

strongly disagree/ disagree/ somewhat disagree/ neither agree nor disagree/ somewhat agree/ agree/ strongly agree

- is reserved,

strongly disagree/ disagree/ somewhat disagree/ neither agree nor disagree/ somewhat agree/ agree/ strongly agree

- is original, comes up with new ideas,

strongly disagree/ disagree/ somewhat disagree/ neither agree nor disagree/ somewhat agree/ agree/ strongly agree

- values artistic, aesthetic experiences,

strongly disagree/ disagree/ somewhat disagree/ neither agree nor disagree/ somewhat agree/ agree/ strongly agree

- has an active imagination,

strongly disagree/ disagree/ somewhat disagree/ neither agree nor disagree/ somewhat agree/ agree/ strongly agree

- is sometimes rude to others,

strongly disagree/ disagree/ somewhat disagree/ neither agree nor disagree/ somewhat agree/ agree/ strongly agree

- has a forgiving nature,

strongly disagree/ disagree/ somewhat disagree/ neither agree nor disagree/ somewhat agree/ agree/ strongly agree

- is considerate and kind to almost everyone,

strongly disagree/ disagree/ somewhat disagree/ neither agree nor disagree/ somewhat agree/ agree/ strongly agree

- does a thorough job,

strongly disagree/ disagree/ somewhat disagree/ neither agree nor disagree/ somewhat agree/ agree/ strongly agree

- tends to be lazy,

strongly disagree/ disagree/ somewhat disagree/ neither agree nor disagree/ somewhat agree/ agree/ strongly agree

- does things efficiently

strongly disagree/ disagree/ somewhat disagree/ neither agree nor disagree/ somewhat agree/ agree/ strongly agree.

**Your circadian rhythm**

Now, let's focus on your preferences and habits related to activity levels at different times of the day. Try to determine whether the following statements are true or not for you. Please indicate your answer by selecting one of the three options:

T - means "yes, true"

? - means "it depends, it's hard to say"

N - means "no, not true"

| I feel I can think the best in the morning. | T/?/N |
| --- | --- |
| I can work equally easily in the day as in the night. | T/?/N |
| If I were to study anything by myself, I would rather do it in the evening. | T/?/N |
| When my usual sleep time comes, I can hardly overcome sleepiness. | T/?/N |
| I like to get up earlier than necessary, e.g. in order to prepare things for the whole day. | T/?/N |
| Regardless of the time of day, I feel almost the same, as to mood and efficiency. | T/?/N |
| I feel drowsy for a long time after awakening. | T/?/N |
| There are moments during the day when I feel unable to do anything. | T/?/N |
| I am usually in an excellent mood in the morning. | T/?/N |
| I can focus at any time of the day, if I have something to do. | T/?/N |
| My work goes better in the afternoon than before noon. | T/?/N |
| I feel uneasy when something disturbs my internal clock, e.g. the change to daylight saving time. | T/?/N |
| I am in my best form in the morning, in the course of the day my energy runs out. | T/?/N |
| At any time of the day, my motivation to work is the same. | T/?/N |
| I feel sluggish in the morning and I warm up slowly during the day. | T/?/N |
| I feel substantial variations of my mood during the day. | T/?/N |

**Your eating habits**

Think back to the past few months. When answering the following questions, take this period of time into consideration.

Over the past few months:

How many times a week did you eat fast food meals or snacks?

- Less than 1 time
- 1-3 times
- 4 or more times

How many servings of fruits did you eat each day?

- 5 or more
- 3-4
- 2 or less

How many servings of vegetables did you eat each day?

- 5 or more
- 3-4
- 2 or less

How many regular sodas or glasses of sweet tea did you drink each day?

- Less than 1
- 1-2
- 3 or more

How many times a week did you eat beans (like pinto or black beans), chicken, or fish?

- 3 or more times
- 1-2 times
- Less than 1 time

How many times a week did you eat regular snack chips or crackers (not low-fat)?

- 1 time or less
- 2-3 times
- 4 or more times

How many times a week did you eat desserts and other sweets (not the low-fat kind)?

- 1 time or less
- 2-3 times
- 4 or more times

How much margarine, butter, or meat fat do you use to season vegetables or put on potatoes, bread, or corn?

- Very little
- Some
- A lot

**Information about You**

Phew... this is the last part of the survey.

Your gender: female/ male

Your year of birth:

What is the population of your place of origin?

- City (with a population of over 500,000 people)
- City (with a population of 200-500 people)
- City (with a population of 50-200 people)
- City (with a population of under 50 people)
- Village (with a population over 1000 people)
- Village (with a population under 1000 people)

What is your father's level of education?

- Incomplete primary education
- Primary education
- Vocational education, incomplete secondary education, or secondary education without a matura exam
- Complete secondary education
- Bachelor's degree, engineering degree
- Master degree
- Doctorate

What is your mother's level of education?

- Incomplete primary education
- Primary education
- Vocational education, incomplete secondary education, or secondary education without a matura exam
- Complete secondary education
- Bachelor's degree, engineering degree
- Master degree
- Doctorate

Do you work for pay?

- No, I do not work for pay
- I work part-time with a flexible schedule.
- I work part-time with a fixed schedule.
- I work full-time with a flexible schedule.
- I work full-time with a fixed schedule.

Do your parent(s) or guardian(s) financially support you?

- I support myself financially.
- Yes, I receive financial support from them.
- Yes, my parents fully support me financially.

People occupy lower or higher positions in society. Below is a scale from 1 to 10, where 1 represents the lowest positions and 10 the highest. Where would you place yourself and your family? 1/2/3/4/5/6/7/8/9/10

Height [cm]

Your current body mass [kg]

Do you currently smoke cigarettes (traditional tobacco)?

- No
- No, but I used to smoke
- Yes

Do you currently smoke e-cigarettes?

- No
- No, but I used to smoke
- Yes

Assess your physical activity. Place yourself on the scale:

- I do not exercise at all 1/2/3/4/5 I do sport intensively 5 times a week

Has a doctor diagnosed you with any of the following chronic diseases? (If you haven’t been diagnosed with any chronic disease, do not select anything; multiple selections are allowed)

- Allergic disease
- Endocrine or metabolic disease
- Gastrointestinal disease
- Immune disease
- Oral cavity disease
- Cardiological disease
- Neurological disease
- Cancer
- Infectious disease

Have you taken psychotropic medications in the past 3 months? Yes/No

Has a doctor diagnosed you with a mental disorder or illness? Yes/No

If “Yes” was selected:

What disorder/illness has a doctor diagnosed you with? (Multiple selections allowed)

- Depressive episode / recurrent depressive disorder
- Anxiety disorders
- Bipolar disorder
- Other:

**Your student ID number**

Just in case, we ask you for it again. Please write down your student ID number, which was assigned to you as a student of the Medical University of Łódź.

**Survey 2**

Dear Student,

Welcome to the next part of the study titled "Assessment of the Relationship Between Selected Health Behaviors and Psychometric Indicators of Mental Health". In this survey, we will ask you to evaluate your sleep, daily problems, physical activity, and more. Completing Survey 2 will take about 20 minutes.

Please write down your student ID number, which was assigned to you as a student of the Medical University of Łódź.

**Sleep**

In the next two sections of the survey, we will ask questions about your sleep during two periods: 1) the past week, and 2) the past 4 weeks.

Sleep in the past week

The following questions relate to your typical sleep habits over the PAST WEEK. Your answers should be as accurate as possible and refer to most of the days and nights during the past week.

What time did you usually go to bed in the evening during the past week?

How much time did it usually take you to fall asleep in the evening during the past week? Please provide your answer in minutes.

What time did you usually get out of bed in the morning during the past week?

On average, how many hours did you actually sleep during the night in the past week (this time may not match the time spent in bed)? Please provide the average sleep time in hours (for example, for 6 hours and 30 minutes, write 6.5).

Please circle the most appropriate answer for each of the following questions. We kindly ask you to answer all the questions.

How often did you sleep poorly during the past week (choose one of the 3 options)...

|  | during the past week, not once. | once or twice during the past week. | three times or more during the past week. |
| --- | --- | --- | --- |
| a) because you couldn't fall asleep within 30 minutes? |  |  |  |
| b) because you woke up in the middle of the night or early in the morning? |  |  |  |
| c) because you had to get up to go to the bathroom? |  |  |  |
| d) because you had trouble breathing? |  |  |  |
| e) because you had a cough or were snoring loudly? |  |  |  |
| f) because you were too cold? |  |  |  |
| g) because you were too hot? |  |  |  |
| h) because you had bad dreams? |  |  |  |
| i) because something was hurting? |  |  |  |
| j) *for other reasons? Please specify them below. |  |  |  |

If you selected point j) "for other reasons" above, please specify them.

You would describe your sleep during the past week as:

- very good
- quite good
- rather bad
- very bad

For each question, choose one of the 3 options:

|  | during the past week, not once. | once or twice during the past week. | three times or more during the past week. |
| --- | --- | --- | --- |
| How often did you take sleeping pills (prescribed by a doctor or available over the counter at a pharmacy)? |  |  |  |
| How often did you have trouble staying alert while driving, during meals, or at social gatherings? |  |  |  |
| How often did you have too little energy to carry out your daily tasks? |  |  |  |

Sleep in the past four weeks

The following questions relate to your typical sleep habits over the PAST FOUR WEEKS. Your answers should be as accurate as possible and refer to most of the days and nights during the past four weeks.

What time did you usually go to bed in the evening during the past four weeks?

How much time did it usually take you to fall asleep in the evening during the past four weeks? Please provide your answer in minutes.

What time did you usually get out of bed in the morning during the past four weeks?

On average, how many hours did you actually sleep during the night in the past four weeks (this time may not match the time spent in bed)? Please provide the average sleep time in hours (for example, for 6 hours and 30 minutes, write 6.5).

Please circle the most appropriate answer for each of the following questions. We kindly ask you to answer all the questions.

How often did you sleep poorly during the past four weeks (choose one of the 4 options)...

|  | during the past week, not once. | once or twice during the past week. | three times or more during the past week. |
| --- | --- | --- | --- |
| a) because you couldn't fall asleep within 30 minutes? |  |  |  |
| b) because you woke up in the middle of the night or early in the morning? |  |  |  |
| c) because you had to get up to go to the bathroom? |  |  |  |
| d) because you had trouble breathing? |  |  |  |
| e) because you had a cough or were snoring loudly? |  |  |  |
| f) because you were too cold? |  |  |  |
| g) because you were too hot? |  |  |  |
| h) because you had bad dreams? |  |  |  |
| i) because something was hurting? |  |  |  |
| j) *for other reasons? Please specify them below. |  |  |  |

If you selected point j) "for other reasons" above, please specify them.

You would describe your sleep during the past four weeks as:

- very good
- quite good
- rather bad
- very bad

For each question, choose one of the 3 options:

|  | during the past four weeks, not once. | once or twice during the past four weeks. | three times or more during the past four weeks. |
| --- | --- | --- | --- |
| How often did you take sleeping pills (prescribed by a doctor or available over the counter at a pharmacy)? |  |  |  |
| How often did you have trouble staying alert while driving, during meals, or at social gatherings? |  |  |  |
| How often did you have too little energy to carry out your daily tasks? |  |  |  |

**Now, a few questions about medications, etc.**

We hope that the questions on the exam today will be better!

Have you used antihistamine allergy medications with sedative/sleep-inducing potential (mainly first-generation antihistamines) during the past four weeks?

- No
- Yes

Have you used systemic antibiotics, antiparasitic medications, or antifungal medications during the past four weeks?

- No
- Yes

Have you been hospitalized during the past four weeks?

- No
- Yes

During the past four weeks, have your chronic illnesses (if you have any) been poorly controlled?

- Not applicable
- No
- Yes

**Your diet**

We are interested in information about your diet during the PAST WEEK.

How many times in the past seven days did you eat fast food meals or snacks?

- Less than once a week
- 1-2 times a week
- 3 times a week or more

How many servings of fruit did you eat per day?

- 1 or fewer servings per day
- 2-3 servings per day
- 4 or more servings per day

How many servings of vegetables did you eat per day?

- 2 or fewer servings per day
- 3-4 servings per day
- 5 or more servings per day

How many glasses of carbonated drinks (such as cola, lemonade, energy drinks) or sweetened tea did you drink per day?

- Less than 1 glass per day
- About 1 glass per day
- More than 1 glass per day

How many times in the past week did you eat legumes, chicken, or fish?

- Once a week or less
- 2-3 times a week
- 4 times a week or more

How many times in the past week did you eat chips, crackers, or similar snacks (regular ones, not low-fat)?

- Less than once a week
- About once a week
- 2 times a week or more

How many times in the past week did you eat desserts or other sweets (regular ones, not low-fat)?

- Once a week or less
- 2-3 times a week
- 4 times a week or more

How much margarine, butter, or other animal fats did you use to season dishes, garnish potatoes or vegetables, or spread on bread?

- Very little
- Quite a small amount
- A little more

How often in the past week did you consume the following products (for each product, choose one of the 6 options)?

|  | I did not consume it at all. | Once a week | 2-3 times a week | 4-5 times a week | Every day | Every day, several times a day. |
| --- | --- | --- | --- | --- | --- | --- |
| Kefir, yogurt, curd, sour cream |  |  |  |  |  |  |
| Hard cheeses, soft cheeses, or cottage cheese |  |  |  |  |  |  |
| Pickled vegetables (cucumbers, cabbage, others) |  |  |  |  |  |  |

**Additional questions about food**

In the past 3 days and today, have you consumed: asparagus, dandelion leaves ("dandelion"), chicory root (found, for example, in coffee substitute), globe artichoke, or Jerusalem artichoke (sunchoke)?

- No
- Yes

Since you have consumed asparagus, dandelion leaves ("dandelion"), chicory root (found, for example, in coffee substitute), globe artichoke, or Jerusalem artichoke (sunchoke) – how much of each vegetable have you consumed in the past 3 days and today?

If you have not consumed a particular vegetable in the past 3 days and today, leave the question unanswered. For the amount of the vegetable you have consumed during this time, express it in any units, such as "a handful," "a full large flat plate," "1 piece," etc.

Asparagus

Dandelion leaves (“dandelion”)

Chicory root (for example, found in coffee substitutes)

Globe artichoke

Jerusalem artichoke (sunchoke)

**How do you feel "in your stomach"?**

Indicate whether you have experienced the following gastrointestinal symptoms in the past 3 days and today (during the period of reporting your meals) – for each symptom, choose one of the 3 options:

|  | No | Yes, to a small extent | Yes, to a significant extent |
| --- | --- | --- | --- |
| Nausea |  |  |  |
| Vomiting |  |  |  |
| Belching |  |  |  |
| A burning sensation in the esophagus or throat |  |  |  |
| Regurgitation of food from the stomach into the esophagus or throat |  |  |  |
| Stomach pain |  |  |  |
| Diarrhea |  |  |  |
| Constipation |  |  |  |
| Bloating (excessive gas accumulation and passing) |  |  |  |
| A feeling of fullness in the abdomen |  |  |  |

**Physical activity**

Please think about all the activities you have performed in the past 7 days at home and in its surroundings, in your academic or professional work, related to moving from one place to another, e.g. commuting to and from work, doing groceries. Also, please consider activities done during your free time, such as walking, recreation, gardening, physical exercises, and sports.

First, we will ask you about activities that require high physical effort, then about activities that require moderate or average effort, and finally about walking and other activities related to walking and sitting.

To begin, please recall all the activities that required intense physical effort, performed during the past 7 days. Intense physical activity causes very fast breathing and a very rapid heart rate. For example, lifting heavy objects, digging soil, aerobics, fast running, or fast cycling require intense physical effort. We are only interested in activities that lasted at least 10 minutes without interruption.

In the past 7 days, have you engaged in activities that required intense physical effort?

- Yes* – For how many days during the past week? Please enter the number of days in the question below.
- No (proceed to question 3)
- I don't know/I'm not sure (proceed to question 3)

If you answered Yes to question 1, please enter the number of days during the past week.

On average, how much time did you spend on activities requiring intense physical effort on such a day? (How many minutes per day?) Or please enter the option "I don't know / I'm not sure."

Now, please recall all the activities requiring moderate (average) physical effort that you performed in the past 7 days. Moderate physical activity leads to slightly faster breathing and a slightly increased heart rate. Examples of moderate physical effort include carrying lighter weights, cycling at a normal pace, playing volleyball, or brisk walking. However, please do not consider walking in general. Again, we are only interested in activities that lasted at least 10 minutes without interruption.

In the past 7 days, have you engaged in activities that required moderate physical effort?

- Yes* – For how many days during the past week? Please enter the number of days in the question below.
- No (proceed to question 5)
- I don't know/I'm not sure (proceed to question 5)

If you answered Yes to question 3, please enter the number of days during the past week when you engaged in activities requiring moderate physical effort.

On average, how much time did you spend on activities requiring moderate physical effort on such a day? (How many minutes per day?) Or please enter the option "I don't know / I'm not sure."

Now, please recall how much time you spent walking in the past 7 days. We are interested in walking related to work, walking on the street (e.g. for shopping, to work), as well as walking for leisure. Again, we are only interested in walking that lasted at least 10 minutes without interruption.

In the past 7 days, did you walk for at least 10 minutes without interruption?

- Yes* – For how many days during that week did you walk for at least 10 minutes without interruption? Please enter the number of days in the question below.
- No (proceed to question 7).
- I don't know / I'm not sure (proceed to question 7).

If you answered Yes to question 5, please enter the number of days during the past 7 days that you walked for at least 10 minutes without interruption.

On average, how much time did you spend walking or on walks during such a day? (How many minutes per day?) Or please enter the option "I don't know / I'm not sure."

Now, how much time did you spend sitting during the past week? This time, please consider only weekdays, i.e., please exclude Saturday and Sunday. Think about activities such as sitting at your desk, sitting during visits with friends, while reading, and also sitting or lying down while watching TV. Please consider the time spent sitting at home, at work, at school/university, in vehicles, and in other places.

Considering the weekdays during the past week, how much time did you typically spend sitting during the day? (How many minutes per day?) Or please enter the option "I don't know / I'm not sure."

**Student ID number**

Please enter your student ID number again. We want to make sure we assign the data correctly to you.

All done? Press "Submit" to send us your answers and complete Survey 2.

**Supplementary Material 2.**

English version of the original tool designed in Polish:

**My meal**

- The "My Meal" form is intended solely for participants in the study "Assessment of the Relationship Between Selected Health Behaviors and Psychometric Indicators of Mental Health" for recording their diet for four days.
- In this form, you need to specify which products and in what quantities you consumed during each meal.
- The form should be filled out and submitted during or immediately after consuming the meal. Alternatively, submitting a series of forms at the end of each day or a single summary form for the day is allowed (see how to report: *link to the instruction*).
- The "My Meal" form classifies food products into 6 categories, which will appear in the following sections of the form:
  1. Meat, cold cuts, fish...
  2. Dairy products and eggs
  3. Cereal products
  4. Vegetables
  5. Fruits and nuts
  6. Sweets
- The amount of food products should be indicated in grams (average weights of single units of products are provided in the pictures for reference). Write the weight only for the products you consumed during the meal; leave the other fields blank. If you consumed a product not listed in this form, simply omit it and do not write it anywhere (see how to estimate food ingredient weights: *link to the instruction*).
- Do not include dietary supplements such as tablets, capsules, etc. (although these are also food).
- Questions about milk, cheese, or creams refer to animal-derived products, not their vegan alternatives.
- Additionally, provide your student ID number at the beginning and end of the form.

***Meat, cold cuts, fish...***

For each category, provide the weight of the product (in grams) that you consumed during the meal. To estimate the weight, use the image below, check on [www.ilewazy.pl](http://www.ilewazy.pl), or weigh it yourself.

1. RED MEAT (beef, pork, lamb, game, liver, etc.). Include red meat in all forms: pieces of meat, chops, cold cuts, sausages, etc.


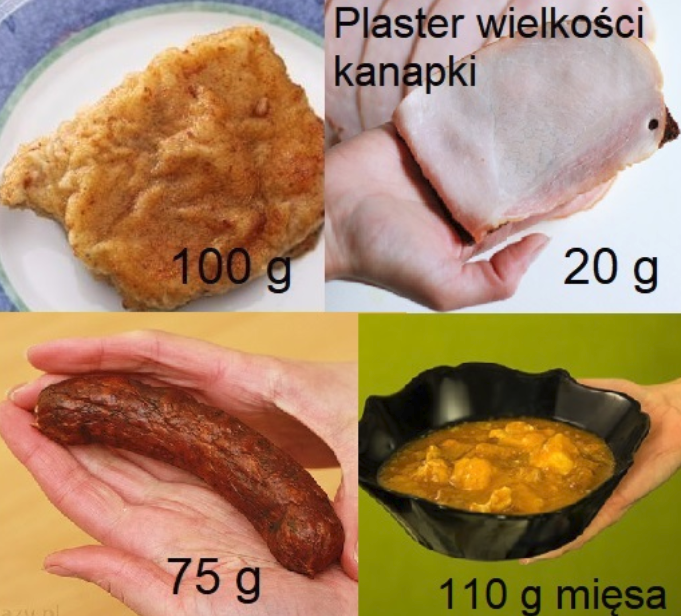


1. WHITE MEAT (poultry). Include white meat in all forms: pieces of meat, chops, cold cuts, sausages, etc.


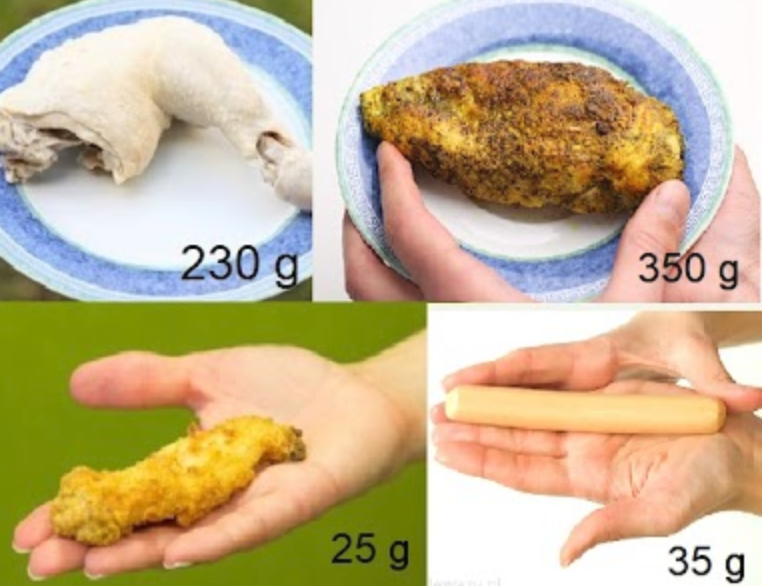


1. FATTY FISH (halibut, salmon, mackerel, sardine, sprat, herring).


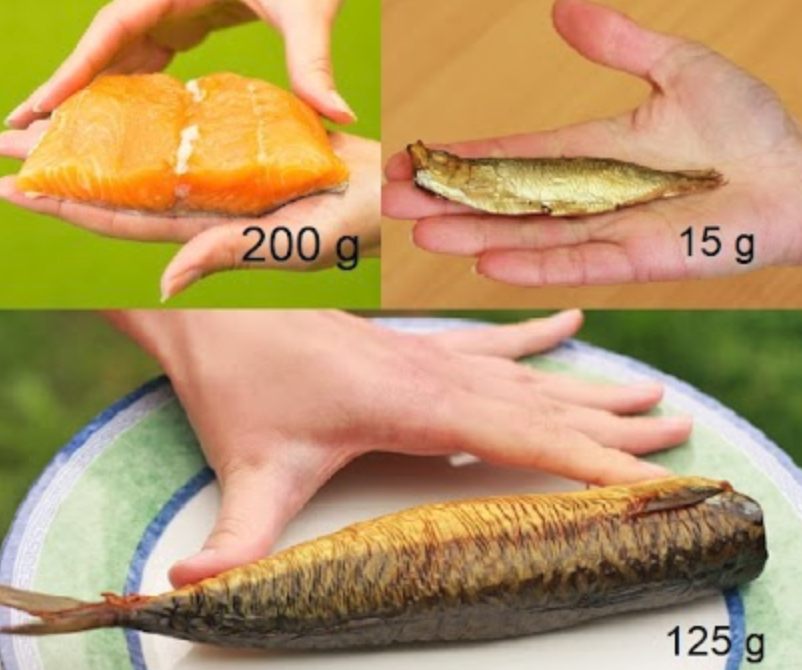


1. OTHER FISH (e.g., cod, trout, perch, etc.).


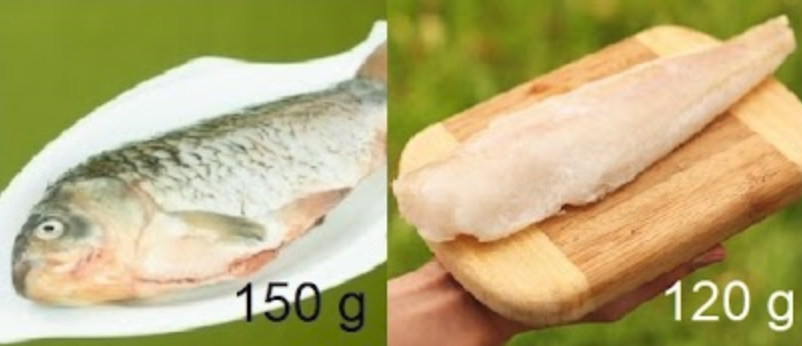


1. FISH OIL AND OTHER FISH OILS.


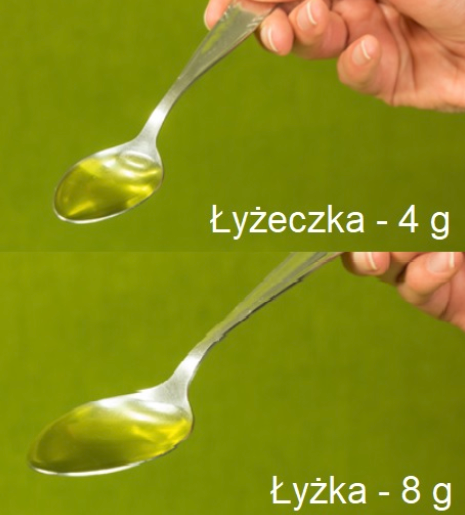


1. SEAFOOD (shrimp, crabs, mussels, oysters, snails, etc.).


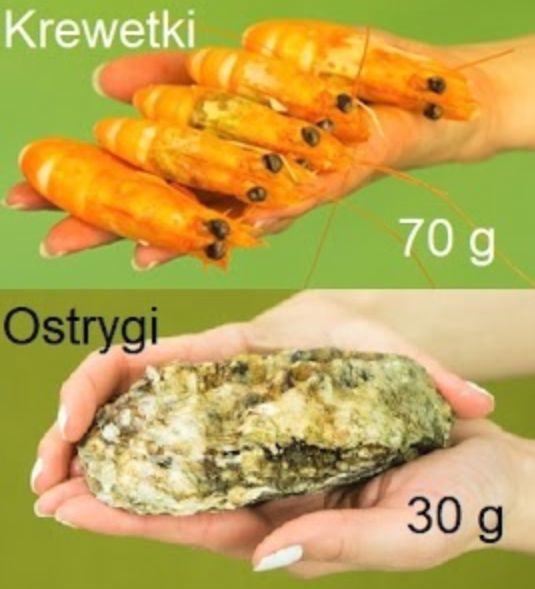


***Dairy Products***

1. MILK (include all types of milk: cow, goat, sheep, etc. – whole, full-fat, semi-skimmed, and skimmed – raw, pasteurized, boiled, and as an ingredient in other dishes). Do not include buttermilk, fermented milk, yogurt, kefir, or cream.


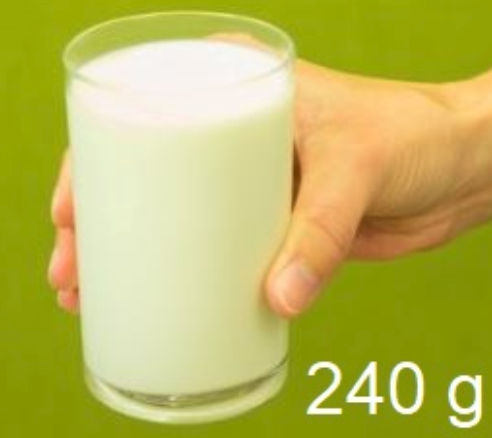


1. WHITE CHEESES (cottage cheese – full-fat, semi-skimmed, and skimmed). Do not include processed cheese, cottage cheese-type products (such as "farmer's cheese"), baked, fried, boiled cheeses, etc.


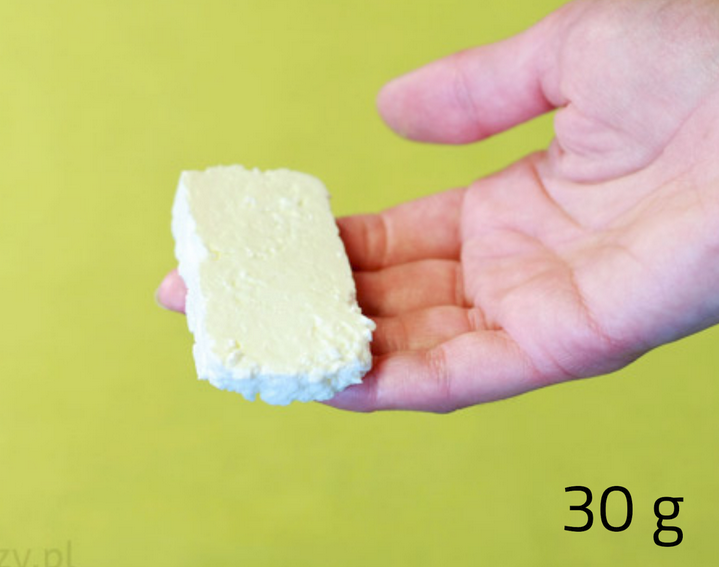


1. YELLOW CHEESES AND OTHERS (hard cheeses – e.g., gouda, cheddar, parmesan, soft cheeses – moldy cheeses, e.g., brie, camembert, and mozzarella). Do not include baked, fried cheeses, or processed cheeses.


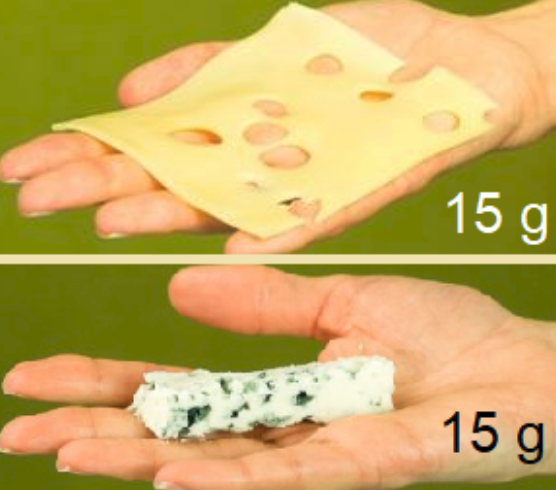


1. YOGURTS, KEFIR, BUTTERMILK (both plain and sweetened, flavored, commercial and homemade, full-fat and low-fat), CREAM (a slightly sour product, do not confuse with cream used in coffee). Do not include heavy cream.


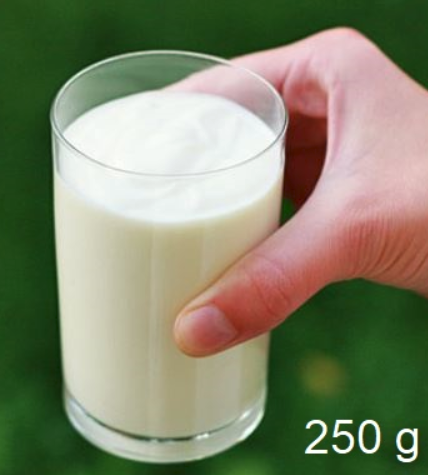


1. EGGS (chicken eggs – in any form)


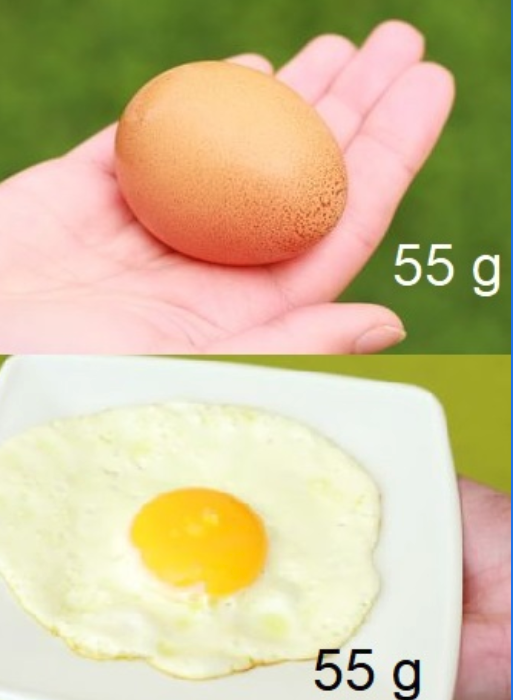


***Cereal Products***

1. WHITE BREAD (white bread, rolls, toast, croissants, etc.)


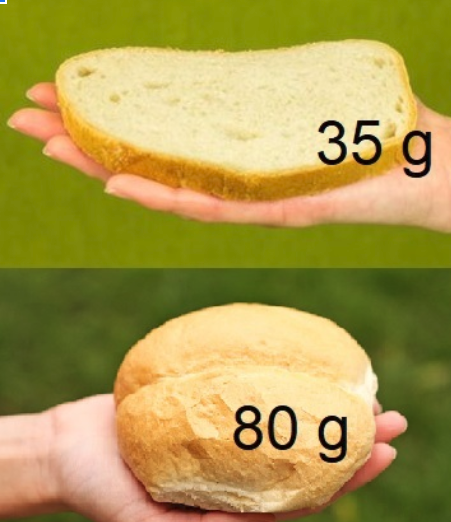


1. WHOLE GRAIN BREAD, GRAHAM BREAD (made from any type of whole grain, unrefined flour)


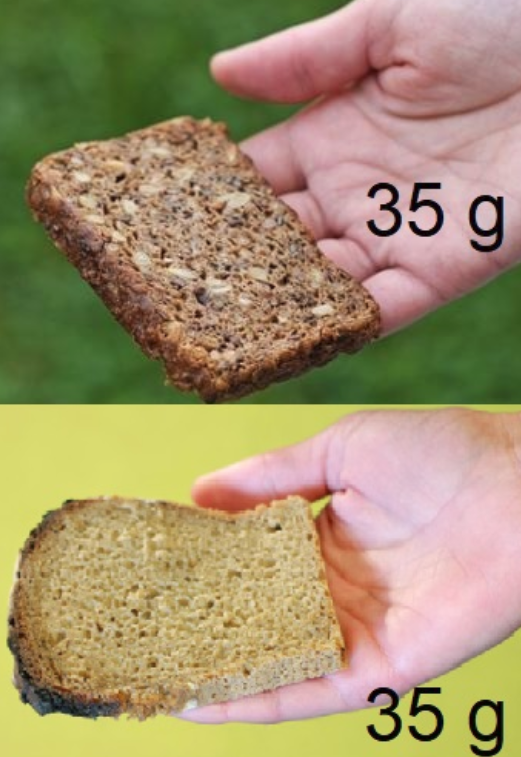


1. GROATS, WHOLE GRAIN PASTA, AND CEREAL FLAKES (rye, wheat, spelled, oat, barley, buckwheat, millet – only whole grain, unrefined products). Provide the weight of cooked products (as shown in the pictures).


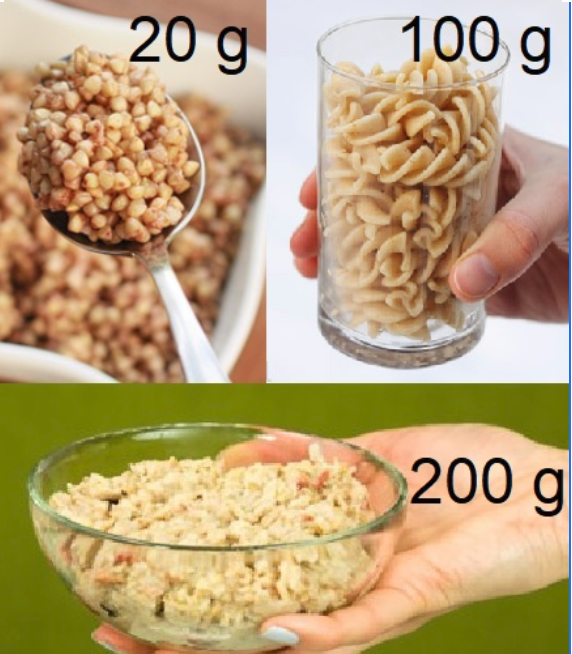


1. MUESLI (raw cereal flakes with added ingredients such as dried fruits, nuts, etc.)


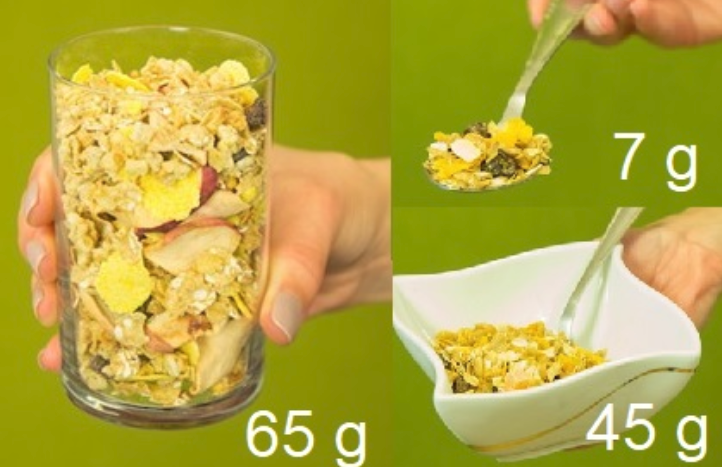


1. WHITE RICE (only white rice – do not include unrefined, brown rice). Provide the weight of cooked rice (as shown in the picture).


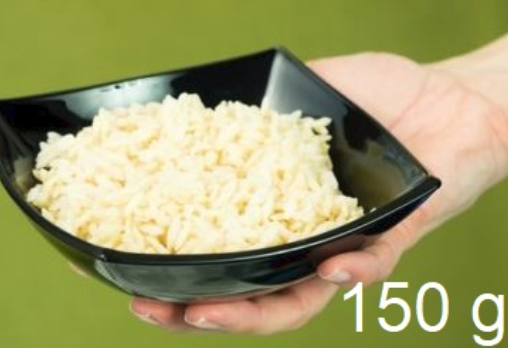


1. KVASS AND UNPASTEURIZED BEER (include only unpasteurized, non-preserved, unfiltered products with a short shelf life – unfortunately, most commercially available products do not meet these requirements).


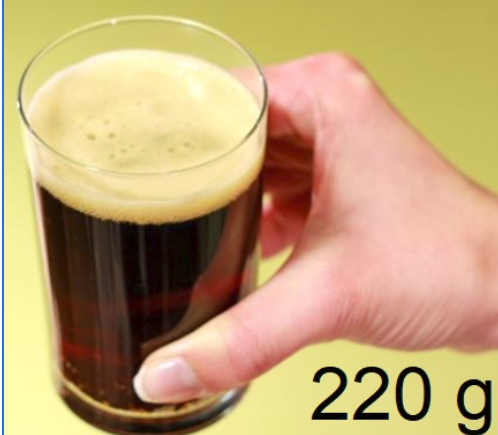


1. WHOLE GRAIN FLOUR, UNREFINED, COARSELY GROUND (rye, wheat, spelt, oat, barley, buckwheat, millet). Only include flour used to prepare products other than the ones listed above to avoid duplication (e.g., other homemade baked goods, flour for thickening dishes). Do not include white, refined flour.


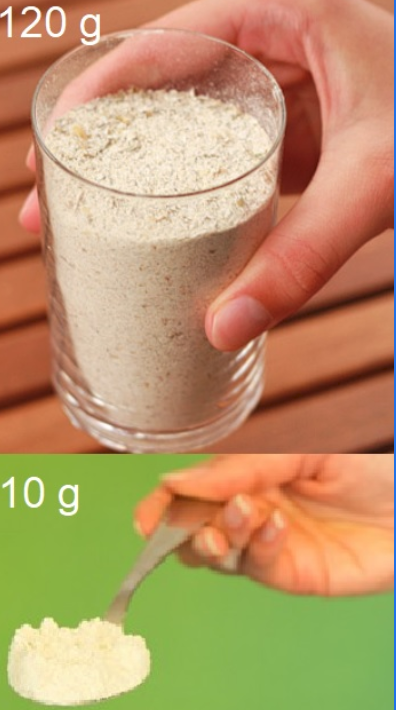


***Vegetables***

1. POTATOES (in all forms)


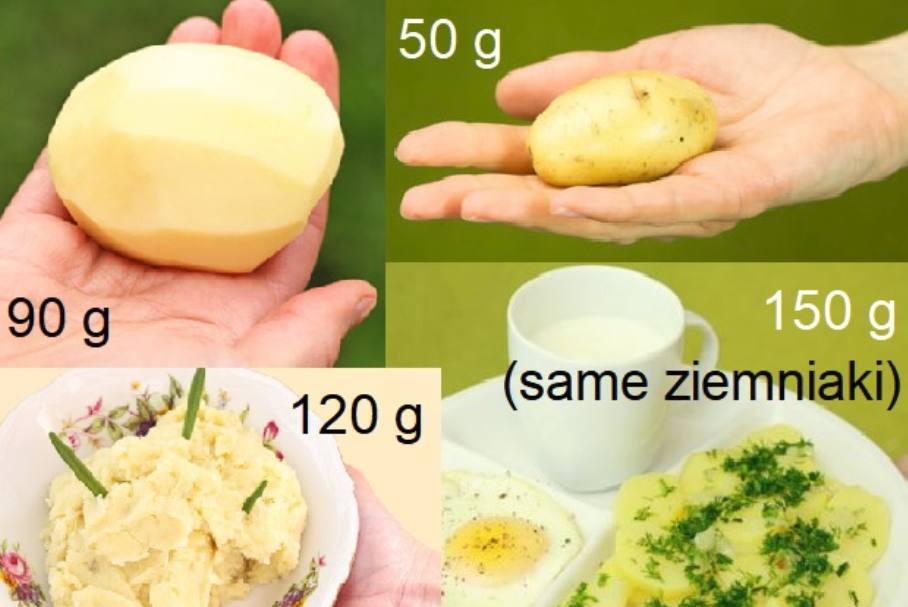


1. CARROT, PARLEY (root), CELERY (root). The weight of the peeled vegetables is provided in the pictures below (peeling reduces the weight by about 25%). Cooking does not significantly affect the weight.


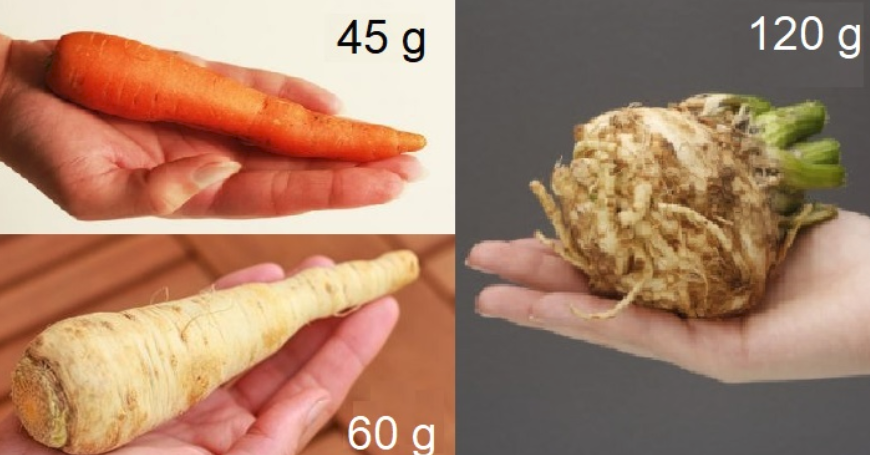


1. BEET. The weight of the peeled vegetables is provided in the pictures below (peeling reduces the weight by about 25%). Cooking does not significantly affect the weight.


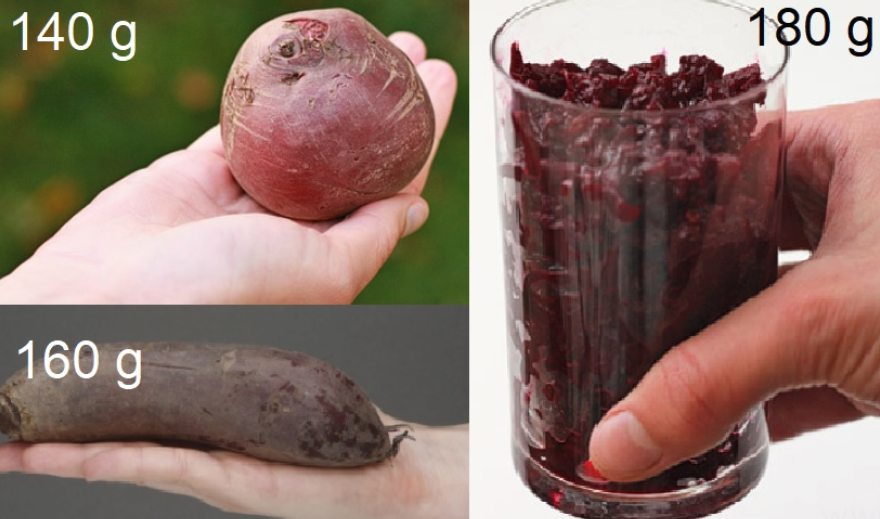


1. CUCUMBER (raw—do not include pickled cucumbers). The pictures below provide the weight of the peeled vegetables (peeling reduces the weight by about 25%).


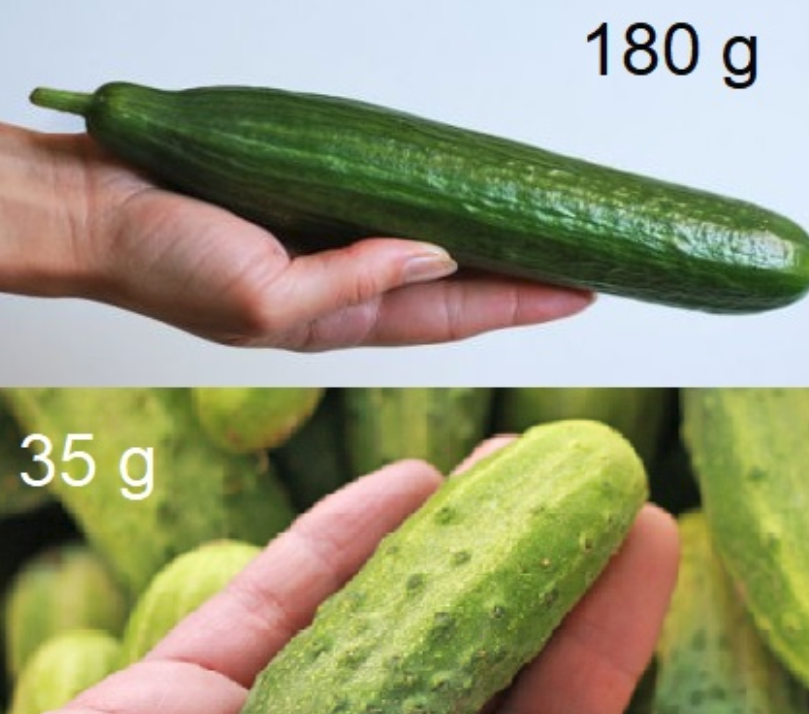


1. PICKLED CUCUMBER AND ITS PICKLING JUICE (only raw pickled cucumbers and the juice – do not include canned cucumbers or cooked pickled cucumbers, e.g., in cucumber soup).


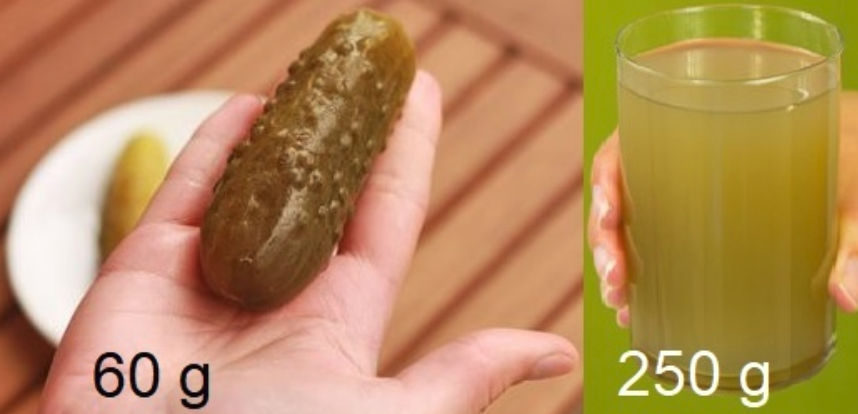


1. CABBAGE (raw and cooked cabbage in any form – do not include sauerkraut).


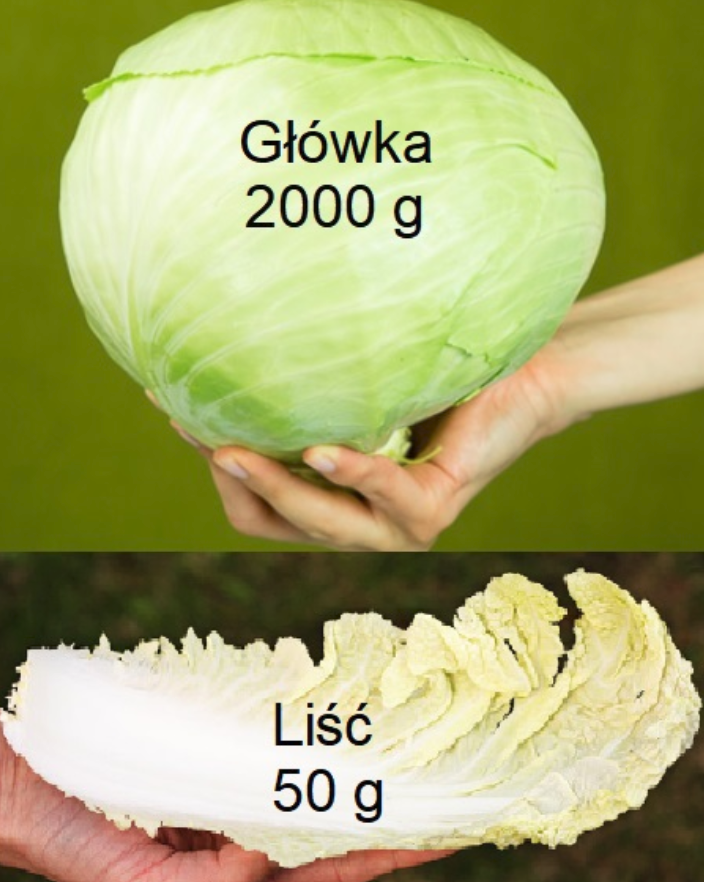


1. SAUERKRAUT AND ITS PICKLING JUICE (only raw sauerkraut and the juice – do not include cooked or sautéed sauerkraut).


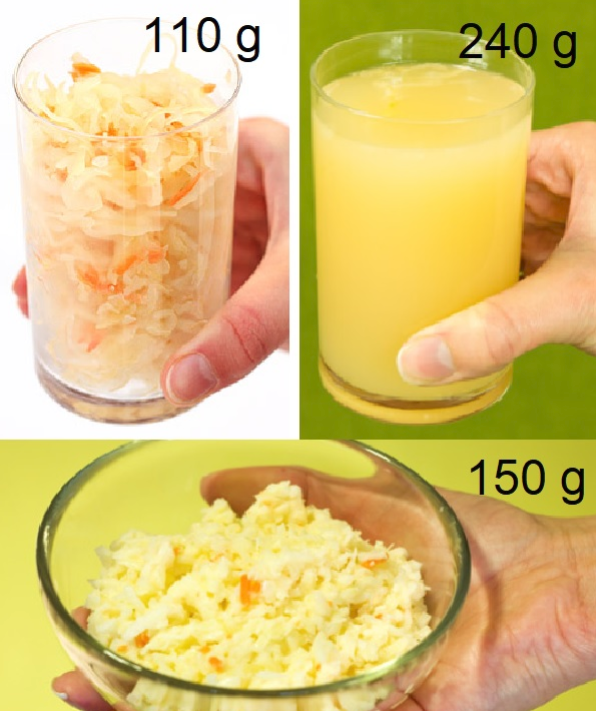


1. OTHER PICKLED VEGETABLES AND THEIR PICKLING JUICE (kimchi, beets, celery, broccoli, etc.). Do not include pasteurized products (many commercially available products are pasteurized).


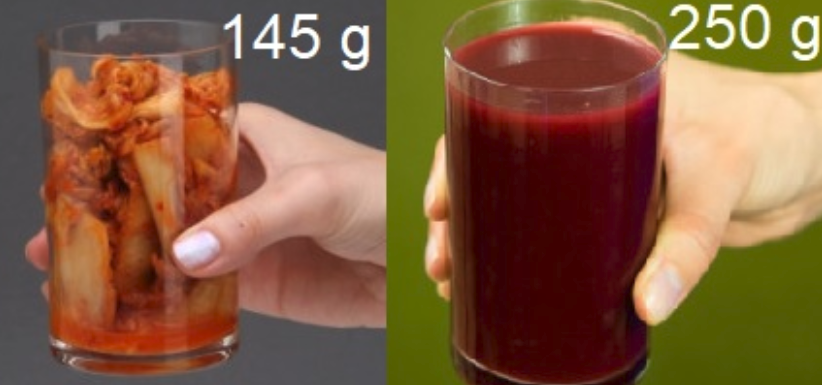


1. ONION, LEEK, GARLIC (in any form).


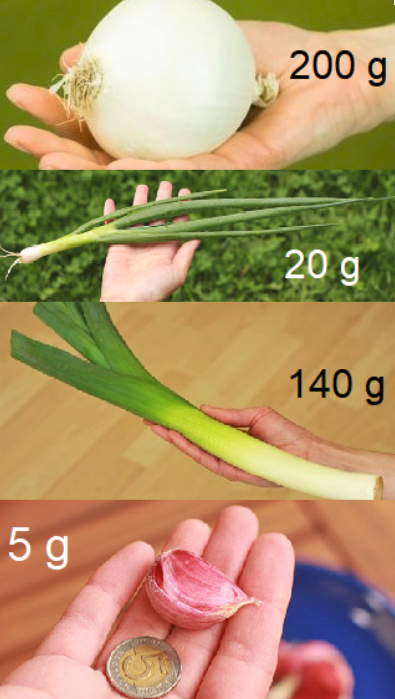


1. LEGUMINOUS VEGETABLES (beans, peas, lentils, soybeans, chickpeas, broad beans – in any form).


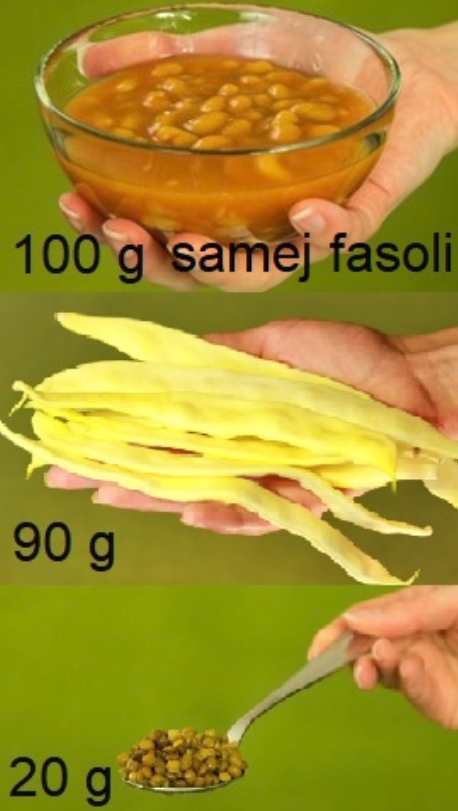


1. ALL OTHER VEGETABLES (in any form - some examples are shown in the picture below).


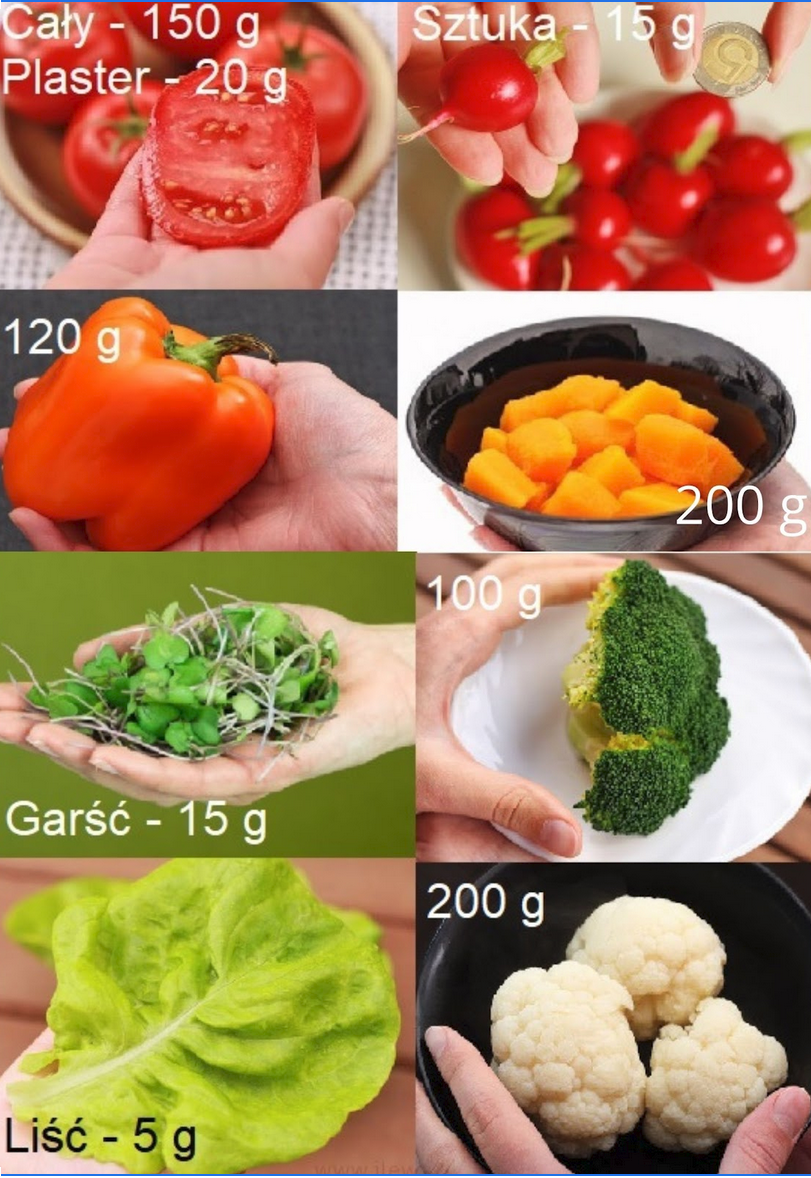


***Fruits and nuts***

1. APPLES (in any form – including juice, but not a beverage).


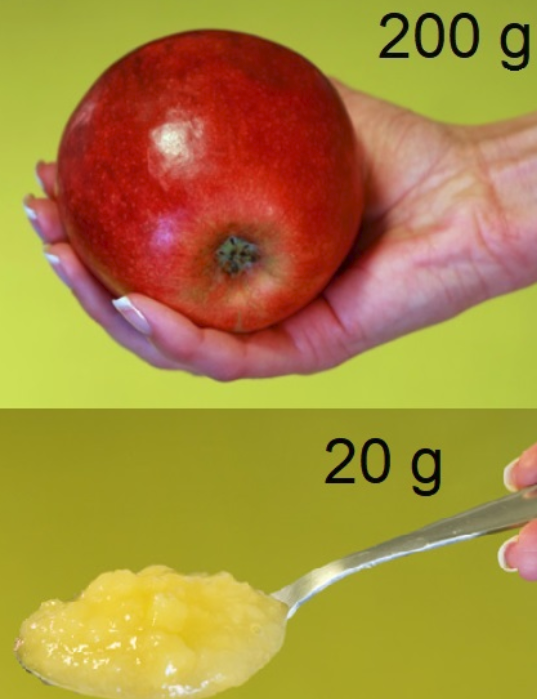


1. CITRUS FRUITS (oranges, tangerines, grapefruits, lemons, etc. – in any form – including juice, but not a beverage). The weight of the peeled fruits is provided in the pictures below (peeling citrus fruits reduces the weight by about 33%).


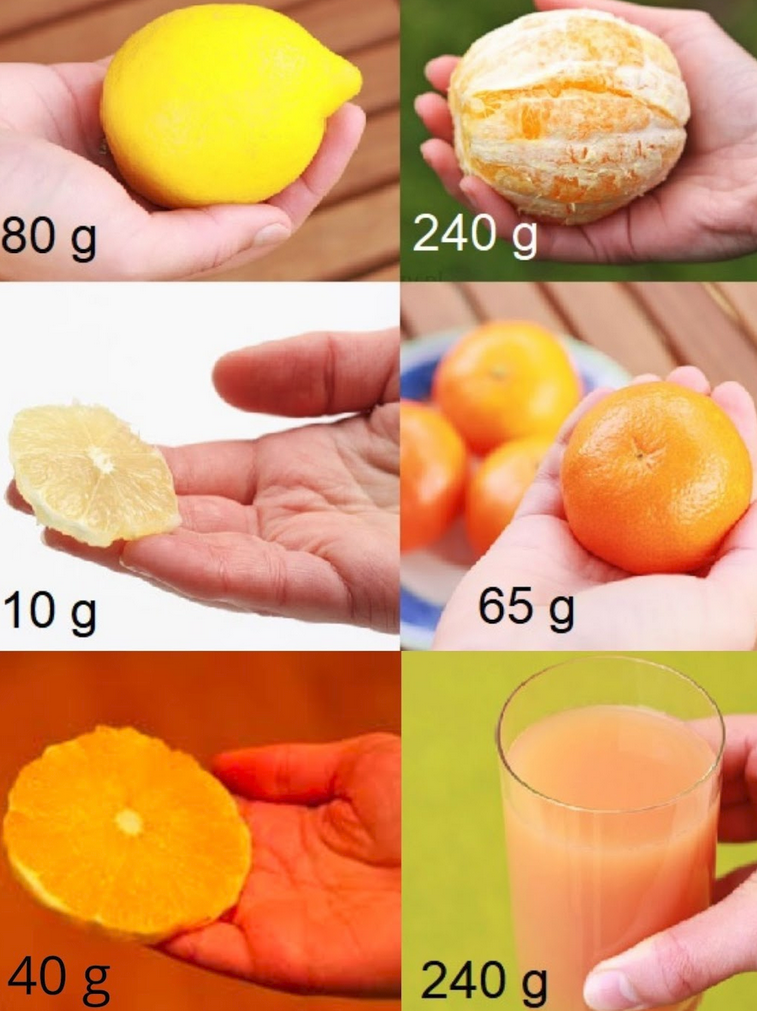


1. BANANAS (in any form). The weight of the peeled banana is provided in the picture below (peeling reduces its weight by about 40%).


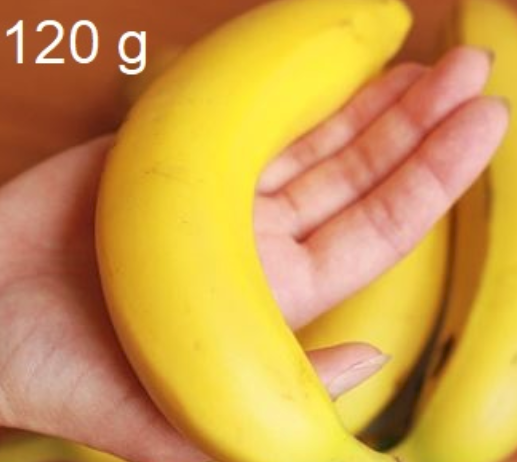


1. ALL OTHER FRUITS (in any form – some examples are shown in the picture below). The weight of the edible parts of the fruits is provided in the pictures below (excluding any pits, cores, skins, etc.).


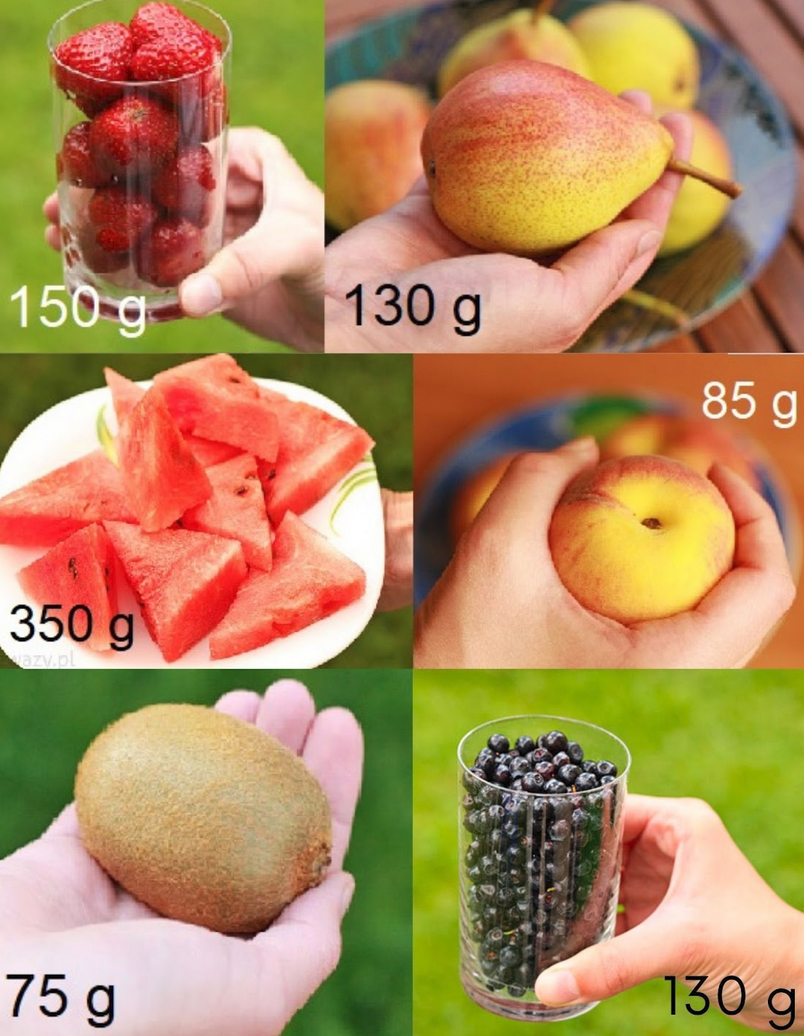


1. NUTS (walnuts, peanuts, cashews, hazelnuts, almonds, pistachios, etc. – in any form – some examples are shown in the picture below).


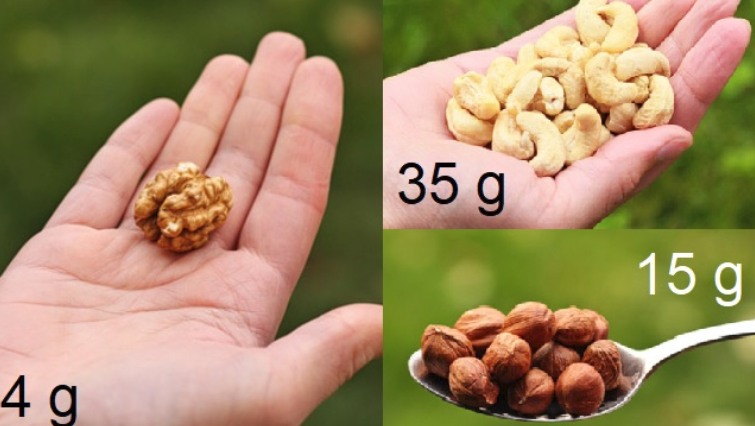


***Sweets***

1. SWEETS WITH LOW/MODERATE SUGAR CONTENT, such as ice cream, cookies, dark chocolate, biscuits, mildly sweet cakes (e.g., pound cake), wafers without chocolate coating, ladyfingers, sweetened breakfast cereals (repeat the entry even if you have already included it in section 3.4. Muesli). Do not include sweets sweetened with xylitol, aspartame, or other sweeteners, but only those containing sucrose or glucose. If you are eating a product with the weight listed on the packaging, enter it. If you have the product label available, classify sweets with a sugar content of less than 45.5 g/100 g of product into this category.


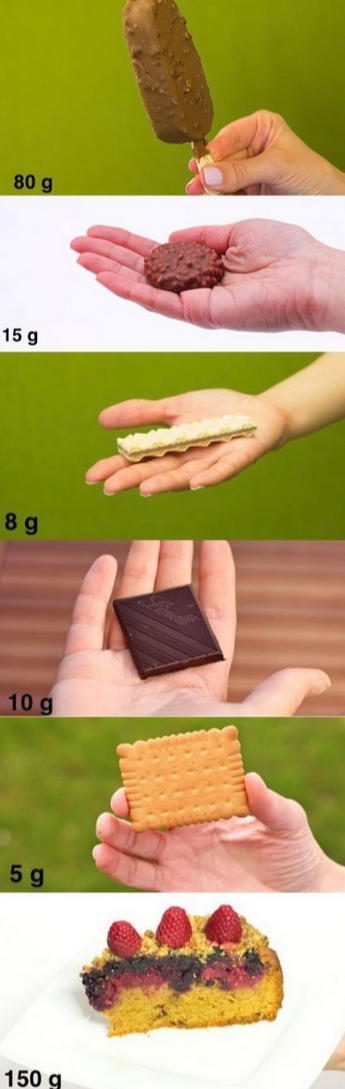


1. SWEETS WITH HIGH SUGAR CONTENT, such as milk chocolate, gummies, jellies, chocolate-covered bars, candies, cakes, and sweet pastries (e.g., brownies). Do not include sweets sweetened with xylitol, aspartame, or other sweeteners, but only those containing sucrose or glucose. If you are eating a product with the weight listed on the packaging, enter it. If you have the product label available, classify products with a sugar content greater than 45.5 g/100 g of product into this category.


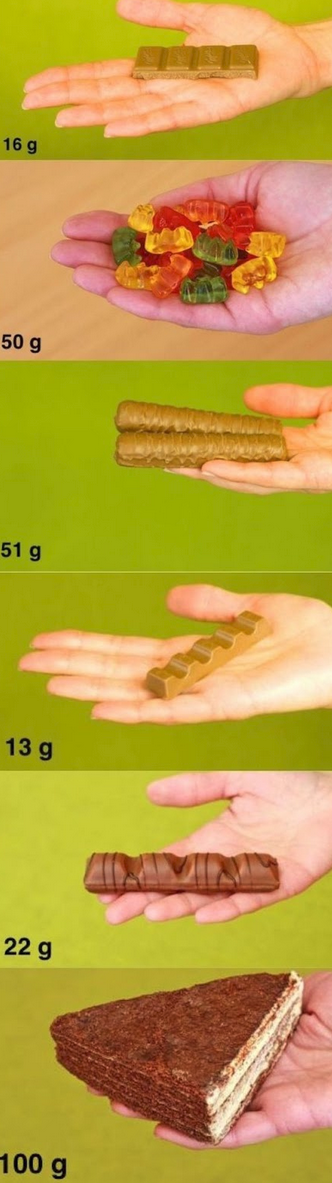


1. SWEETENED BEVERAGES – provide the weight of the beverage. Do not include "sugar-free" beverages.


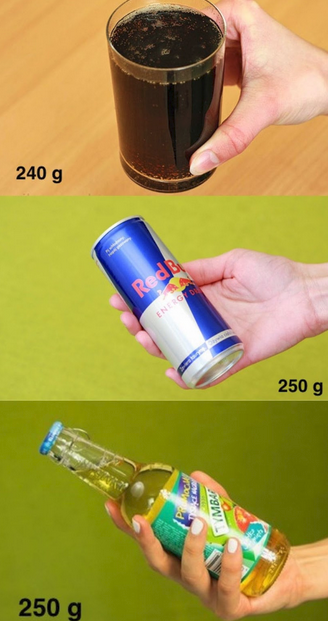


1. SUGAR OR HONEY – provide the weight of the sugar/honey itself – in any form (on bread, eaten "straight from the jar," added to tea/coffee, etc., including artificial honey), unless it has already been included in previous sections of the form. Do not include xylitol, erythritol, or other sweeteners.


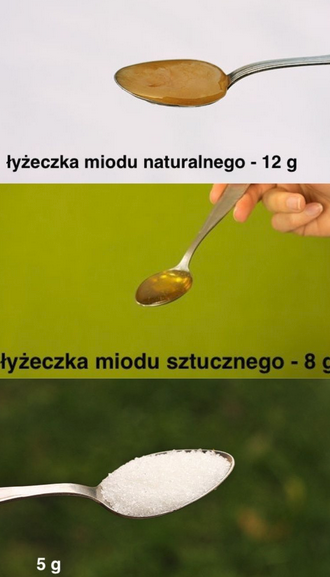


THE END

**Supplementary Material 3.**

Association between the fermented food consumption and the presence of GI symptoms in four days preceding the exam.

Table 5. Association between the fermented food consumption and the presence of GI symptoms in four days preceding the exam. GI = gastrointestinal.

^a^Adjusted for sex, socioeconomic status, BMI, suffering from a mental health illness, suffering from any chronic disease, physical activity (IPAQ), general diet quality (STC), current cigarette smoking/use, each of five personality traits (BFI-S).

^b^Intensity of GI symptoms in 0-20 scale, where the bigger the number, the more intense the symptom is and “0” stands for lack of any symptoms.

| **GI symptoms** | **Tetriles of fermented food consumption [grams]** | | | | | |
| --- | --- | --- | --- | --- | --- | --- |
|  | Linear trend | | | | | |
|  | Raw analysis | | | Adjusted analysis^a^ | | |
|  | Contrast estimate | | | Contrast estimate | | *p*-value |
|  | Point | 95% CI | *p*-value | Point | 95% CI |  |
| Intensity of GI symptoms | | | | | | |
| Intensity of GI symptoms^b^ | -0.49 | -1.44, 0.45 | 0.3048 | -0.73 | -1.64, 0.19 | 0.1196 |
| Particular GI symptoms: | | | | | | |
| Nausea | -0.06 | -0.23, 0.12 | 0.5336 | -0.07 | -0.25, 0.11 | 0.4408 |
| Vomiting | 0.01 | -0.03, 0.05 | 0.6504 | 0.02 | -0.02, 0.06 | 0.4204 |
| Belching | -0.08 | -0.25, 0.09 | 0.3822 | -0.10 | -0.28, 0.07 | 0.2337 |
| Heartburn | -0.09 | -0.22, 0.04 | 0.1977 | -0.09 | -0.22, 0.04 | 0.2040 |
| Regurgitation | -0.06 | -0.18, 0.07 | 0.4004 | -0.09 | -0.22, 0.04 | 0.1815 |
| Abdominal pains | -0.08 | -0.27, 0.11 | 0.4217 | -0.13 | -0.31, 0.06 | 0.1836 |
| Diarrhea | -0.04 | -0.21, 0.11 | 0.5806 | -0.04 | -0.21, 0.12 | 0.6044 |
| Constipation | 0.07 | -0.05, 0.19 | 0.2441 | 0.05 | -0.07, 0.17 | 0.4575 |
| Flatulence | -0.08 | -0.28, 0.12 | 0.4396 | -0.12 | -0.32, 0.08 | 0.2635 |
| Feeling of fullness | -0.09 | -0.28, 0.10 | 0.3598 | -0.15 | -0.34, 0.05 | 0.1531 |
|  | Non-linear trend | | | | | |
|  | Raw analysis | | | Adjusted analysis^a^ | | |
|  | Contrast estimate | | *p*-value | Contrast estimate | | *p*-value |
|  | Point | 95% CI |  | Point | 95% CI |  |
| Intensity of GI symptoms | | | | | | |
| Intensity of GI symptoms^b^ | -0.49 | -2.32, 0.95 | 0.4103 | -0.21 | -1.82, 1.40 | 0.7953 |
| Particular GI symptoms: | | | | | | |
| Nausea | -0.12 | -0.43, 0.19 | 0.4458 | -0.06 | -0.38, 0.26 | 0.7342 |
| Vomiting | 0.01 | -0.06, 0.08 | 0.7939 | 0.04 | -0.04, 0.11 | 0.3716 |
| Belching | -0.12 | -0.43, 0.18 | 0.4377 | -0.05 | -0.36, 0.26 | 0.7435 |
| Heartburn | 0.04 | -0.19, 0.27 | 0.7265 | 0.08 | -0.16, 0.32 | 0.5147 |
| Regurgitation | 0.01 | -0.21, 0.23 | 0.9370 | 0.04 | -0.18, 0.27 | 0.7085 |
| Abdominal pains | -0.23 | -0.56, 0.10 | 0.1812 | -0.13 | -0.46, 0.20 | 0.4305 |
| Diarrhea | -0.15 | -0.43, 0.13 | 0.2891 | -0.11 | -0.40, 0.18 | 0.4516 |
| Constipation | 0.05 | -0.16, 0.26 | 0.6354 | 0.04 | -0.17, 0.25 | 0.7092 |
| Flatulence | -0.10 | -0.45, 0.25 | 0.5726 | -0.04 | -0.39, 0.32 | 0.8373 |
| Feeling of fullness | -0.07 | -0.41, 0.27 | 0.6864 | -0.02 | -0.37, 0.32 | 0,9023 |
